# A new measure of group decision-making efficiency

**DOI:** 10.1186/s41235-020-00244-3

**Published:** 2020-09-17

**Authors:** Cheng-Ju Hsieh, Mario Fifić, Cheng-Ta Yang

**Affiliations:** 1 Department of Chemical Engineering, National Cheng Kung University, Tainan, Taiwan; 2 Department of Psychology, Grand Valley State University, Allendale, MI USA; 3 Department of Psychology, National Cheng Kung University, Tainan, Taiwan; 4 Institute of Allied Health Sciences, National Cheng Kung University, No. 1, University Road, Tainan, Taiwan 701

**Keywords:** Systems factorial technology, Workload capacity, Group decision-making

## Abstract

It has widely been accepted that aggregating group-level decisions is superior to individual decisions. As compared to individuals, groups tend to show a decision advantage in their response accuracy. However, there has been a lack of research exploring whether group decisions are more efficient than individual decisions with a faster information-processing speed. To investigate the relationship between accuracy and response time (RT) in group decision-making, we applied systems’ factorial technology, developed by Townsend and Nozawa (*Journal of Mathematical Psychology 39*, 321–359, 1995) and regarded as a theory-driven methodology, to study the information-processing properties. More specifically, we measured the workload capacity *C*_*AND*_(*t*), which only considers the correct responses, and the assessment function of capacity *A*_*AND*_(*t*), which considers the speed-accuracy trade-off, to make a strong inference about the system-level processing efficiency. A two-interval, forced-choice oddball detection task, where participants had to detect which interval contains an odd target, was conducted in Experiment 1. Then, in Experiment 2, a yes/no Gabor detection task was adopted, where participants had to detect the presence of a Gabor patch. Our results replicated previous findings using the accuracy-based measure: Group detection sensitivity was better than the detection sensitivity of the best individual, especially when the two individuals had similar detection sensitivities. On the other hand, both workload capacity measures, *C*_*AND*_(*t*) and *A*_*AND*_(*t*), showed evidence of supercapacity processing, thus suggesting a collective benefit. The ordered relationship between accuracy-based and RT-based collective benefit was limited to the *A*_*AND*_(*t*) of the correct and fast responses, which may help uncover the processing mechanism behind collective benefits. Our results suggested that *A*_*AND*_(*t*), which combines both accuracy and RT into inferences, can be regarded as a novel and diagnostic tool for studying the group decision-making process.

## Significance

Previous studies have shown the so-called collective benefit. That is, performance is more accurate when participants work as a group in which they can communicate with each other verbally or non-verbally, and with an exchange of decision evidence or internal estimate of confidence. However, it is still unclear whether group decisions are more efficient than individual decisions since a tradeoff may exist between speed and accuracy. In other words, increasing the number of group members may increase the group’s response accuracy, but at the same time would slow down the processing speed. The aim of the study was to learn about the relationship between accuracy and response-time (RT) measures in group decision-making. To sum up, our results replicated the previous findings that showed the collective benefit for accuracy: the group’s detection sensitivity was higher than the best individual’s detection sensitivity only when group members’ detection sensitivities were similar. The measures of processing speed, the workload capacity measures, revealed that group decision-making was of supercapacity processing. In addition, our results suggested that the assessment function of workload capacity, which combines both accuracy and RT into inferences, can be regarded as a novel and diagnostic tool to study the group decision-making process. The current study is not only a replication of the previous studies, but also highlights the importance of combined accuracy and RT measures in the inference of the group decision-making process.

## Introduction

An old saying goes, “Two heads are better than one.” Combining group members’ opinions to make a coherent decision is usually regarded as a better means of decision-making than having an individual make a decision alone (Clemen, 1989). Many important real-world decisions are collective decisions, such as juries rendering verdicts or a team of radiologists reading X-rays. The situation in which group decisions are considered to be superior to individual decisions is termed “wisdom of crowds”^1^ (Surowiecki, 2004).

In the literature, the primary focus has been on learning about the mechanism that underlies group decisions. Previous studies have investigated the properties of cooperation between two or more participants in perceptual decision tasks (Bahrami, Olsen, Latham, Roepstorff, Rees, & Frith, 2010; Bahrami, Olsen, Bang, Roepstorff, Rees, & Frith, 2012a; Sorkin & Dai, 1994; Sorkin, Hays, & West, 2001; Sorkin, West, & Robinson, 1998) and found that a group exhibits a decision advantage—a so-called “collective benefit”—over an individual decision-maker. There are several proposed possible explanations for the existence of the collective benefit.

First, it is possible that the collective benefit results from a reduced workload, as group members strategically split information among themselves. The fact that each member can focus attention on a subset of information (which means the group does not have to focus its attention on the entirety of information) could lead to an increase in collective processing efficiency. However, group decision accuracy may be limited by individual ability because the group must rely on the capabilities of each member. This possibility has been challenged and ruled out by Barr and Gold (2014). Barr and Gold (2014) manipulated the group size (one to four members) and the quantity of information (partial or full) that each member received. Their results showed that groups viewing the entirety of information significantly outperformed groups whose members viewed limited portions of information and suggested that strategically splitting information does not necessarily lead to a collective benefit.

Second, the collective benefit could be due to the statistical facilitation effect (Green & Swets, 1966; Lorge & Solomon, 1955; Sorkin & Dai, 1994; Sorkin et al., 2001; Swets, Shipley, McKey, & Green, 1959). In stochastic modeling, adding more independent random variables to a parallel processing system can lead to a faster and more accurate task completion. The statistical facilitation that is achieved through the redundancy gain, has been used by engineers to decrease failure rate of their machines. Analogously, one can demonstrate a similar effect in human group decision-making, by increasing the number of independent decision-makers who work in parallel. The overall group’s achievement will be better than that of any individual member working alone. Interestingly, the collective benefit, that is due to the statistical facilitation and the redundancy gain, is merely an outcome of the statistical improvement – that is, the group benefit is not achieved by the group members’ interaction. Such a statistical facilitation effect is conditioned on the use of the so-called first-termination rule,^2^ which means that the system would wait for the fastest and correctly responding unit to complete and would then use it to make the final decision while ignoring the unfinished or incorrectly responding units.

Third, the collective benefit could be a result of the integration of evidence collected by each group member via social interaction (Bahrami et al., 2010; Lorenz, Rauhut, Schweitzer, & Helbing, 2011). This explanation is different from the first two in that it assumes the collective sum of knowledge occurs not only as a simple sum of individual knowledge, but as novel knowledge created through a series of social interactions. As a result, the performance of a group is better than that of the best observer or exceeds the expectations of individual members working in isolation (Collins & Guetzkow, 1964; Davis, 1969, 1973). Consistent with the coactive model^3^ (Houpt & Townsend, 2011; Schwarz, 1989, 1994; Townsend & Nozawa, 1995), the collective benefit may have occurred because the individual contribution is weighted and integrated into a single information channel following a “weighted-and-sum” principle of information integration.

Recently, an increasing number of studies has challenged the idea that group decisions would always outperform individual decisions. For example, Fific and Gigerenzer (2014) suggested that adding more decision-makers does not necessarily enhance the group performance. The best individual may match the collective decision accuracy or even outperform the group, especially when free riders exist. In addition, a single expert, under certain conditions, can outperform the group (e.g., Gordon, 1924; Graham, 1996; Winkler & Poses, 1993). The debate over the potential negative effect of collaboration on decision-making has intensified work that explores the conditions under which the collective benefit/cost may arise.

A potential factor that may influence the collective effect was performance similarity between group members. In a two-interval forced-choice oddball detection task in which participants had to decide which interval contained an odd target, Bahrami et al. (2010) investigated whether participants can utilize their partner’s confidence rating to improve group decision sensitivity.^4^ The results showed that only in a consistent group, in which the two group members had similar detection sensitivity, was the group decision superior to individual decisions. Specifically, the authors used *S*_*min*_ and *S*_*max*_ to represent the detection sensitivity of the worse and better individual, respectively, and only when *S*_*min*_/*S*_*max*_ ≥ 0.4 did the group show the collective benefit, i.e., *S*_*dyad*_/*S*_*max*_ > 1 (S_dyad_ denotes group detection sensitivity). By contrast, in an inconsistent group (i. e. , *S*_*min*_/*S*_*max*_ < 0.4), the group decision was worse than the decision made by its better group member. Bahrami et al. (2010) further suggested that the results supported the weighted confidence-sharing model (WCS), which assumes that individuals can take advantage of the confidence information, i.e., an internal estimate of the probability of being correct; the final decision is made based on the weighting function of the group members’ confidence. The WCS model can be considered a variant of the coactive models.

Another important factor in understanding the collective is the tradeoff between accuracy and speed in group decision-making (see Heitz, 2014 for a review). The collective effect can be measured by both response accuracy and RT. However, when used in the same task, the two measures can have an inverse relationship. That is, increasing the number of group members may increase the group’s response accuracy, but, at the same time, could create longer RTs; for example, as the group size increases, group members require more time to communicate with each other to reach a consensus. Thus, it is reasonable to speculate that collaboration can increase response accuracy but slow down the decision RT. The above-mentioned studies focused mainly on the effect of collaboration on accuracy measures (e.g., Bahrami et al., 2010, 2012a, b) while neglecting its effect on the measure of processing speed.

Two other studies utilized RT measures (e.g., RT distribution analysis) to assess the collective effect (e.g., Brennan & Enns, 2015; Yamani, Neider, Kramer, & McCarley, 2017). Brennan and Enns (2015) tested the violation of the race-model inequality to infer the collective benefit. In general, the race model assumes that two processing units are racing to reach a certain decision criterion (Miller, 1982). The two units work independently, and the faster unit, which reaches the decision criteria first, will determine the response outcome. In our domain of interest, the units are defined as the individual decision-makers. The race-model inequality assumes that two group members work independently and in parallel (simultaneously). In a nutshell, a violation of the race-model inequality would suggest that the two decision-makers did not work independently of each other and that at some point, prior to making a final decision, they interacted with each other. In terms of modeling processing systems, this situation is defined by coactive processing. Using a visual enumeration task in which participants were required to count the number of targets (0/1/2) presented against the distractors, Brennan and Enns (2015) demonstrated that the observed RT data violated the race-model inequality, thereby supporting the notion that the two decision-makers did not work independently and collaboration would facilitate decision RTs.

Using another RT measure, i.e., workload capacity, Yamani et al. (2017) examined how collaboration affects individual processing efficiency in terms of the information-processing speed. Workload capacity is a measure of the change in processing efficiency (speed) at the individual subject level when the system’s workload (i.e., the number of decision-makers) increases, as proposed by the framework of Systems Factorial Technology (SFT, Little, Altieri, Fific, & Yang, 2017; Townsend & Nozawa, 1995). According to SFT, increasing the number of processing units (i.e., the system’s workload) can have three different effects on the processing speed of an individual processing unit. In the case of limited-capacity processing, the speed of processing per processing unit slows down when more units operate at the same time. In the case of unlimited-capacity processing, the speed of processing per processing unit remains unchanged when more processing units are added. In the case of supercapacity processing, the speed of processing per processing unit speeds up by the addition of more decision units. This could be the result of a facilitatory interaction between decision units. It is notable that an unlimited-capacity system implies that the efficiency of an individual unit remains unchanged, whereas the system overall can be performing better compared with the individual unit, due to stochastic considerations. In the context of group decision-making, the limited capacity indicates some form of inhibition between individual decision-makers when they work as a group. In the case of unlimited capacity, the addition of more group members does not affect individual efficiency. In the case of supercapacity, efficiency improves as a result of the group members’ facilitatory interaction. Yamani et al. (2017) study adopted the shared-gaze technique which allows participants to see where their partner is looking in order to study whether collaboration can benefit scan in teams. Results showed supercapacity processing when both group members were required to find and respond to a target; by contrast, limited capacity was found when the faster searcher found and responded to the target. The supercapacity results suggested that, by holding fixation on the target, the faster searcher can cue their partner to the target location, which, in turn, boosts the processing speed of the slower searcher. The limited-capacity results suggested that shared gaze offered no benefits but slowed down the processing for the faster searcher.

To summarize, collective benefit/cost can be measured by either response accuracy or RTs. These two measures play complementary roles in understanding the mechanism underlying group decisions. However, to our knowledge, there is no prior study that combines accuracy and RT measures to infer the dynamic process of group decision-making. This raises several related questions. Are the inferences from the two measures consistent enough to draw similar conclusions? Is it possible to use a single performance index to quantify the collective effect by considering the two measures simultaneously? In the present study, we integrated, within one study, the two approaches by applying SFT (Little et al., 2017; Townsend & Nozawa, 1995). Before we go into the details about the present study, we first briefly introduce the theory and methodology of Systems Factorial Technology (SFT).

### Systems Factorial Technology

SFT (Little et al., 2017; Townsend & Nozawa, 1995) is a useful tool for analyzing and diagnosing the dynamic decision-making process. A wide range of fields in cognitive research have utilized SFT, such as visual search (Zehetleitner, Krummenacher, & Müller, 2009), memory search (Townsend & Fific, 2004; Van Zandt & Townsend, 1993), face perception (Ingvalson & Wenger, 2005; Yang, Altieri, & Little, 2018), classification (Fific, Nosofsky, & Townsend, 2008), change detection (Yang, 2011; Yang, Chang, & Wu, 2013; Yang, Hsu, Huang, & Yeh, 2011), cued detection (Yang, Little, & Hsu, 2014; Yang, Wang, Chang, Yu, & Little, 2019), word processing (Houpt, Sussman, Townsend, & Newman, 2015; Houpt, Townsend, & Donkin, 2014), audiovisual processing (Altieri & Yang, 2016; Yang et al., 2018; Yang, Yu, & Chang, 2016), and group decision-making (Yamani et al., 2017). According to SFT, two important information-processing properties of group decision-making can be uncovered, such as a group’s organization during task participation (i.e., how two individuals work together to achieve a group decision) and workload capacity (i.e., individual decision efficiency varies as a function of the number of decision-makers).

In this paper, we used the workload capacity measure to quantify group decision-making efficacy. The workload capacity measures can be used to indicate the amount and type of the potential collective benefit. Here, we introduced two types of capacity measures. First is the AND capacity^5^ (*C*_*AND*_(*t*)), a standard measure of workload capacity, developed by Townsend and colleagues (e.g., Townsend & Nozawa, 1995), which considers only the RT data of correct responses. The AND capacity is analyzed by comparing the group processing efficiency to a baseline predicted from the UCIP model (i.e., unlimited-capacity, independent, parallel model), which assumes that all group members work independently and in parallel. The workload capacity is formalized as a ratio of the cumulative reverse hazard functions, *K*(*t*) = ln *F*(*t*) where *F*(*t*) = P (RT ≤ *t*) (Chechile, 2003, 2011; Townsend & Eidels, 2011; Townsend & Wenger, 2004), and is expressed as:

1 $$ {C}_{AND}(t)=\frac{K_1(t)+{K}_2(t)}{K_{12}(t)} $$

for *t* > 0, where *K*_1_, *K*_2_, and *K*_12_ represent the cumulative reverse hazard function of the two non-collaborative conditions, in which participants perform the task independently, and the collaborative condition, in which participants work together with social interaction (here, the non-verbal communication), respectively. The interpretation of *C*_*AND*_(*t*) > 1 implies that group performance is better than the prediction from the UCIP model—that is, the system engages in supercapacity processing. When *C*_*AND*_(*t*) = 1, it suggests an unlimited-capacity processing system, implying that individual decision performance is unchanged when the number of group members increases. When *C*_*AND*_(*t*) < 1, it suggests a limited-capacity processing system, implying that social interaction may, in fact, even slow down individual decision time.

Second, we introduced a new measure, assessment function of workload capacity *A*_*AND*_(*t*), to analyze group decision efficiency. To our knowledge, *A*_*AND*_(*t*) has not been used to study the group decision-making process. Similar to *C*_*AND*_(*t*), *A*_*AND*_(*t*) has the advantage of inferring the dynamic processing efficiency as a function of RT (Donkin et al., 2014; Townsend & Altieri, 2012). Better than *C*_*AND*_(*t*), *A*_*AND*_(*t*) combines both accuracy and RT data into the analysis, such that we can analyze the decision efficiency of four response conditions: (a) correct and fast, (b) correct and slow, (c) incorrect and fast, and (d) incorrect and slow. The inferences of *A*_*AND*_(*t*) were similar to *C*_*AND*_(*t*); the details of the data analysis and inferences will be introduced in the “Data analysis” section. Please see Table 1 for the SFT-related theoretical glossary. More details can also be found in Townsend and Altieri (2012).

**Table 1:** Systems Factorial Technology (SFT)-related theoretical glossary

Assessment function	Assessment function of workload capacity combines both accuracy and response time (RT) into analysis. It can be used to infer the processing efficiency of four response conditions: (a) correct and fast, (b) correct and slow, (c) fast and incorrect, and (d) incorrect and slow
Coactive models	A parallel architecture which assumes that inputs from parallel channels are combined into a common accumulator. A decision is made when the total activation reaches the decision criterion
Detection sensitivity	A maximum slope of the psychometric function. The steeper the slope, the higher the detection sensitivity
Race-model inequality	The race models assume that two decision units are racing to reach a decision criterion. If race models hold, the survivor function for the collaborative condition is bounded below by a combination of survivor functions from the two non-collaborative individual conditions. Violation of this bound implies that two decision units may interact with each other with supercapacity processing; that is collective benefit
Statistical facilitation	The RT or accuracy gain produced by the standard parallel model
Stopping rules	Rules to determine when a system stops processing, special cases of interest are self-termination and exhaustive rules
Systems Factorial Technology	A theory-driven methodology that emphasizes identification of organization of processes through manipulation of experimental factors, typically under the assumption of factorial selectivity
Unlimited-capacity, independent parallel model	An architecture which assumes that each decision unit in a system work in parallel. The efficiency of the system does not change as the number of decision units increases
Workload capacity	A theoretical construct pertaining to influences on processing speed performance when the number of decision units of a system (i.e., the number of decision-maker in the present context) is varied

### The present study

In the present study, we conducted two psychophysics experiments by collecting both accuracy and RT data to test whether collaboration through the exchange of confidence information can promote group decision efficiency. In the first experiment, we extended the study by Bahrami et al. (2010) by adopting the two-interval forced-choice oddball detection task. In the second experiment, we used a yes/no Gabor detection task. Both accuracy-based and time-based measures were computed to infer the collective effect. First, the accuracy-based collective effect was computed by comparing the dyad’s sensitivity with the maximum individual sensitivity (i.e., *S*_*dyad*_/*S*_*max*_). Of particular interest was testing whether this effect would change as a function of relative detection sensitivity between the two group members (i.e., *S*_*min*_/*S*_*max*_). Second, the time-based collective effect was inferred from two workload capacity measures (*C*_*AND*_(*t*)) and *A*_*AND*_(*t*))) as introduced in the previous section.

Our first goal of the study is to replicate the effect of sensitivity similarity on joint decisions. We expect that only when group members have similar detection sensitivities, the collective benefit would be observed; that is, group detection sensitivity will be higher than the detection sensitivity of the best observer. Our second goal is to evaluate how RT and accuracy measures are consistent enough to draw similar conclusions about the collective effect. We expect to observe significant correlations between the time-based and accuracy-based measures of collective effect. Our last goal is to establish the assessment function of workload capacity as a standard measure of group decision efficiency. Considering the speed-accuracy trade-off effect, *A*_*AND*_(*t*), which combines both accuracy and RT into the analysis, can be regarded as a better index for quantifying the group decision advantage.

### Experiment 1

In Experiment 1, a two-interval forced-choice oddball detection task was adopted. The relative detection sensitivity between the group and the best observer was computed to infer the accuracy-based collective effect. Additionally, the workload capacity of group decision-making was assessed as a time-based measure of the collective effect. If both accuracy-based and time-based measures can reflect the collective benefit, we should expect that the accuracy-based collective effect (i.e., *S*_*dyad*_/*S*_*max*_) is greater than 1 and that time-based measures reveal supercapacity results.

## Method

### Participants

Fourteen (eight male and six female; age: 21.3 ± 2.05 years) undergraduate students at National Cheng Kung University volunteered to participate in this experiment and were randomly divided into seven pairs. All of the participants were right-handed. Before the experiment, participants signed an informed consent form. The ethics approval for the study was obtained from the Human Research Ethics Committee of National Cheng Kung University, and the experiment was conducted in accordance with the approved guidelines and regulations. The participants were either compensated in the amount of NTD 140 per hour or received class credit for their participation.

### Equipment

A desktop computer with a 3.20 G-Hz Intel Core i7–8700 Processor, Intel UHD Graphics 630, and 8 GB of RAM controlled the display and recorded the manual responses. Stimuli were presented on a 19-in. CRT monitor with a refresh rate of 75 Hz and a display resolution of 1024 x 768 pixels. The viewing distance was kept at 60 cm and a chin-rest was used to prevent any head movements. The experiment was programmed using Psychtoolbox (http://psychtoolbox.org/) from MATLAB (Mathworks Inc., Natick, MA, USA).

### Design, stimuli, and procedure

**Fig. 1:**
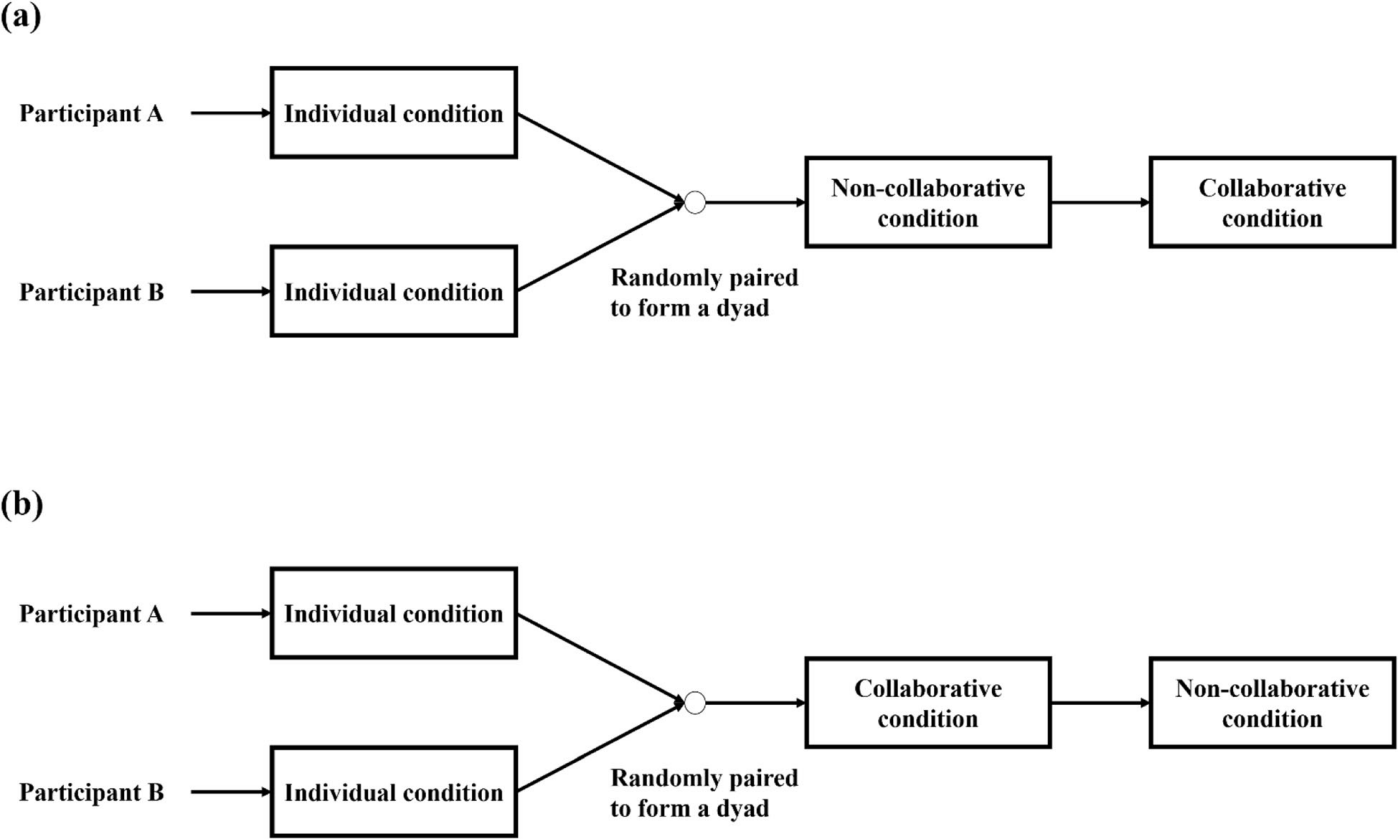
A flowchart of the task design. **a** the non-collaborative condition first. **b** the collaborative condition first

**Fig. 2:**
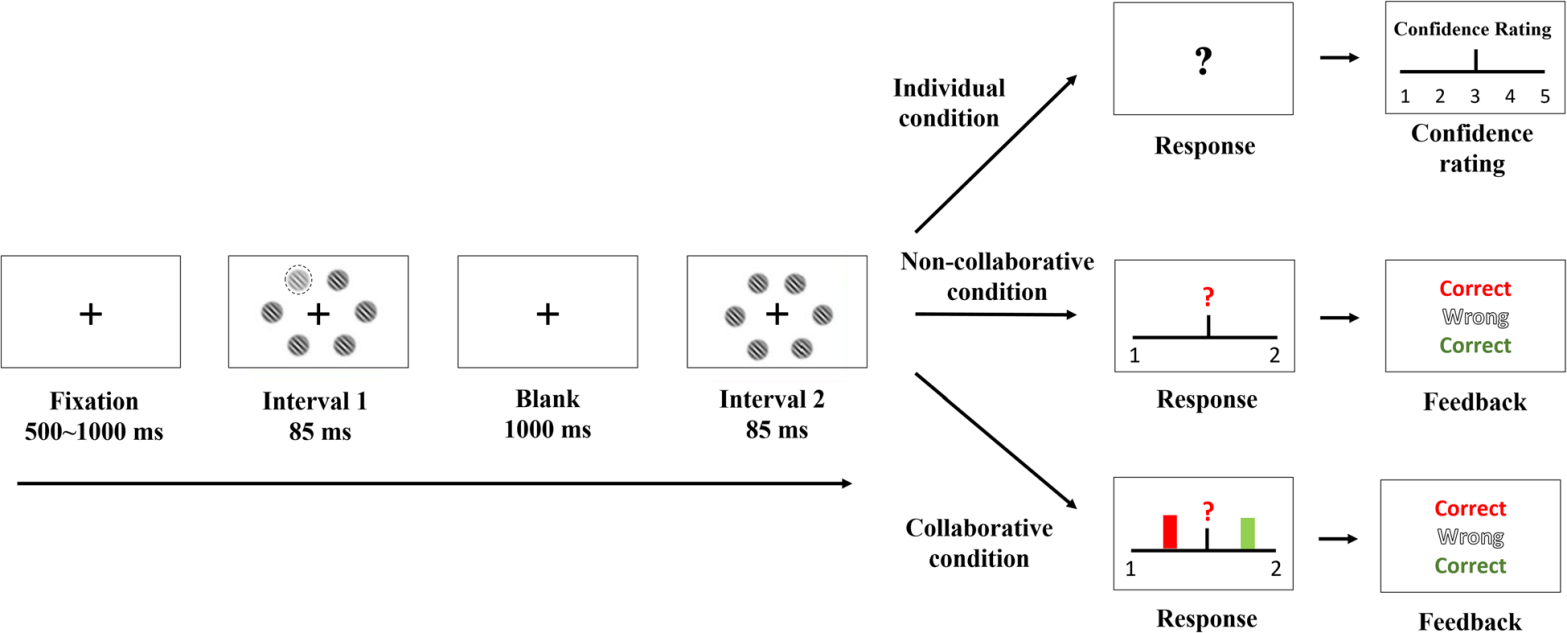
An illustration of the trial procedure in Experiment 1

Figure 1 shows the flowchart of the task design and Fig. 2 shows an illustration of the trial procedure for each condition.^6^ All the participants participated in three cooperation conditions: the individual condition, the non-collaborative condition, and the collaborative condition. Participants first performed the individual condition. Then they were randomly paired to form a dyad.^7^ As a dyad, each participant faced their screen and was positioned next to their partner at a distance of 55 cm. They could only see their partner’s responding hand. Therefore, we believe that other forms of communication (e.g., body language) are not available. The order of the collaborative and non-collaborative conditions was counterbalanced across dyads (see Fig. 1).

The response mode was slightly different across the three cooperation conditions. In the individual condition, participants were required to complete the task alone and to provide a response by clicking the left or right button of the mouse to indicate which interval contained the odd target. After they had made their decision, they were asked to rate their confidence in their judgment using a Likert-type scale from 1 (“very doubtful”) to 5 (“absolutely sure”). The confidence information was used for the collaborative condition in the next stage.

In the non-collaborative condition, two participants working as a dyad performed the task together but without any communication. One participant delivered a response with a mouse click while the other participant responded with a keyboard press. During each trial, only one of the participants was required to respond and the person who was required to respond was dependent on the color of the question mark, with the color green indicating that the participant with the keyboard should respond and the color red indicating that the participant with the mouse should respond. The color (green/red) was randomly selected with equal probability. See Fig. 2 for an example, because the question mark is colored in red, the participant with the mouse should respond and the participant with the keyboard does not need to respond. After a response was made, a feedback display was shown to indicate the correctness of the present decision (middle) and the correctness of the participants’ individual decision about the same trial tested in the individual condition (top and bottom colored in red and green, respectively).

In the collaborative condition, the procedure was the same as that in the non-collaborative condition except that each participant’s confidence information was presented on a horizontal line to indicate the participants’ confidence in the judgment regarding the same trial that was tested in the individual condition. Participants could only communicate by exchanging their confidence information and no other forms of communication (e.g., verbal communication) were allowable. Specifically, the confidence information was represented by two colored marks (i.e., red and green marks representing the confidence rating made by the two participants, respectively). The farthest-left side represented the fact that the participants were very confident that the target was presented at Interval 1, while the farthest-right side represented the fact that the participants were very confident that the target was presented at Interval 2. This display of the participants’ confidence allowed them to communicate with each other and non-verbally exchange their confidence ratings.

Each trial started with a fixation cross displayed for a random duration of between 500 and 1000 ms. Afterward, two consecutive stimulus displays were presented for 85 ms with a 1000-ms blank interval in between the two displays. Each display contained six Gabor patches, which were placed in an imaginary circle with a radius of 232.7 pixels (8.0°). All the Gabor patches were placed equidistant from each other. Each Gabor patch was vertically oriented (standard deviation of the Gaussian envelope: 0.45°; spatial frequency: 1.049 cycle/°). In one of the two displays, there were an odd target and five distractors; in the other display, there were six identical distractors. The contrast of the distractor was 10%. The contrast of the target was 1.5, 3.5, 7.0, or 15.0% higher than the distractor (i.e., the contrast level of the target was 11.5, 13.5, 17.0, or 25.0%) and was randomly selected from one of the four contrast levels. The background was colored in gray with a luminance of 36.34 cd/m^2^. The participant’s task was to make a two-interval forced-choice (2IFC) to indicate which interval contained the odd target, as accurately and quickly as possible after the question mark was presented. The question mark was presented until the participants delivered a response or 2 s had elapsed.

Each cooperation condition involved two sessions in order to collect enough data points. In each session, participants first performed a practice block of 64 trials and then 10 blocks of formal trials. Each block consisted of 2 (target was presented at Interval 1 or 2) × 4 (contrast levels of the target) × 8 (trials per combination).

### Data analysis

The practice trials were excluded from the analysis. Correct RTs within a range of the 2.5% quartile and the 97.5% quartile were extracted for further analysis to exclude the outliers. To compute the collective effect, we compared the data of the collaborative condition (i.e., two participants working together with an exchange of the partner’s confidence) to the data of the non-collaborative condition (i.e., two participants working independently without any communication). In doing that, we were able to conclude that the collective benefit stems from the non-verbal communication rather than from the social facilitation effect. It is notable that, in the following, the term “non-collaborative-individual” represents the individual performance extracted from the non-collaborative condition rather than from the individual condition. The reason why we did not directly compare the data of the collaborative condition to the data of the individual condition is that a large processing difference existed between the two conditions. That is, in the collaborative and non-collaborative conditions, participants were required to first process the color of the question mark such that they would know who was going to respond in the present trial, whereas there was no need to process the color of the question mark in the individual condition. If the processing was less efficient in the collaborative condition than it was in the individual condition, we did not know whether the results were due to collective cost or whether making a group decision would require an additional process.

First, we conducted a mixed-design analysis of variance (ANOVA) to test the differences in accuracy and mean correct RTs, with the social condition (better observer, worse observer, collaboration) serving as a between-subject factor and with contrast level (11.5, 13.5, 17.0, or 25.0%) serving as a within-subject factor. It is notable that the data of the worse observer and better observer was extracted from the data of the non-collaborative condition and that the collaboration data was extracted from the data of the collaborative condition. We did not incorporate the data of the individual condition into analysis, as mentioned above; however, the data would provide necessary information for the collaborative condition.

The participants’ perceptual sensitivity was derived from response accuracy. To quantify perceptual sensitivity, the maximum slope of the psychometric function of contrast sensitivity was estimated for individuals and dyads, respectively (Bahrami et al., 2010, 2012a, b). We fitted the data with a cumulative Gaussian function to estimate the maximum slope of the psychometric function. A larger slope indicates higher sensitivity. The cumulative Gaussian function with two parameters, i.e., bias (*b*) and variance (*σ*^2^), was fitted by a procedure of maximum likelihood estimation via the MATLAB Palamedes toolbox (http://www.palamedestoolbox.org/) (Mathworks Inc., Natick, MA, USA). The psychometric curve, denoted as *P*(*∆c*) where *∆c* is the contrast difference between the target and distractor, can be expressed as:

2 $$ P\left(\Delta  c\right)=H\left(\frac{\Delta  c+b}{\sigma}\right) $$

where *H*(*z*) is the cumulative Gaussian function:

3 $$ H(z)\equiv {\int}_{-\infty}^z\frac{dt}{{\left(2\pi \right)}^{\frac{1}{2}}}{e}^{\frac{-{t}^2}{2}} $$

Here, *P*(*∆c*) corresponds to the probability of responding that the odd target was presented during the second interval. By definition, the variance *σ*^2^ is related to the maximum slope of the psychometric curve, denoted as *s*, which can be expressed as:

4 $$ s=\frac{1}{{\left(2\pi {\sigma}^2\right)}^{\frac{1}{2}}} $$

We defined “collective effect” as the ratio of the dyad’s slope (*S*_*dyad*_) to that of the better observer (i.e., the individual with a larger slope, *S*_*max*_). A collective effect larger than 1 indicates that the dyad managed to gain an advantage over its better individual, suggesting collective benefit. Values below 1 indicate that collaboration was counterproductive and that the dyad did worse than its better individual—namely, collective cost. The effect of relative detection sensitivity on the collective benefit can be revealed by plotting the collective effect against the relative sensitivity between the worse and the better observer (*S*_*min*_/*S*_*max*_).

Moreover, we adopted SFT to calculate the workload capacity of group decision-making (Little et al., 2017; Townsend & Nozawa, 1995). We used the SFT R package (Houpt, Blaha, McIntire, Havig, & Townsend, 2014) to compute workload capacity *C*_*AND*_(*t*) and the assessment function of workload capacity *A*_*AND*_(*t*) (please see the introduction for more details). Notably, *A*_*AND*_(*t*) incorporates both RT and accuracy data into the analysis. The probabilities of responses can be classified into four categories: (a) the probability that a correct response is made by time *t* (correct and fast decision), (b) the probability that an incorrect response is made by time *t* (incorrect and fast decision), (c) the probability that a correct response will be made but it has not happened by time *t* (correct and slow decision), and (d) the probability that an incorrect response will be made but it has not happened by time *t* (incorrect and slow decision).

The influence of additional decision-makers was measured for each of the four response types by comparing the collaborative performance with the predicted UCIP baseline derived from the non-collaborative condition. This interpretation is more nuanced than the standard capacity coefficient. In contrast to the standard capacity coefficient *C*_*AND*_(*t*), when interpreting the *A*_*AND*_(*t*) function, one must consider the type of response being made. The following examples may help readers understand how we make inferences using *A*_*AND*_(*t*). The interpretation of *A*_*AND*_(*t*) for correct and fast responses bears the closest resemblance to the standard capacity coefficient. When *A*_*AND*_(*t*) = 1 for correct and fast responses, the implication is that the observed responses made before time *t* are as probable as expected by the UCIP baseline model. A correct and fast *A*_*AND*_(*t*) > 1 means that participants make more correct responses by time *t* than expected and, thus, exhibit a form of supercapacity. Similarly, correct and fast *A*_*AND*_(*t*) < 1 implies that fewer correct responses are made by time *t* than expected by the UCIP model (i.e., capacity is limited). The interpretation differs for the other types of responses. For example, for the incorrect and slow responses, *A*_*AND*_(*t*) > 1 indicates that more incorrect responses are made after time *t* than is expected by the UCIP model, which implies a form of limited capacity.

Finally, a quantitative comparison was made to observe the relationship between the accuracy-based measure and time-based measure. We first employed functional principal components analysis (fPCA) with varimax rotation to decompose *C*_*AND*_(*t*) and *A*_*AND*_(*t*) into several principal components (Burns, Houpt, Townsend, & Endres, 2013). The SFT R package was adopted (Houpt, Blaha, et al., 2014). fPCA is a structural extension of standard PCA (Ramsay & Silverman, 2005) and can be used to describe the entire functions using a small number of scalar values (i.e., the loading of the principal component) (Burns et al., 2013). The approach enabled us to describe which part of the function-level property is crucial for distinguishing collective effect as a function of RT. Then, we tested the relationship between the factor scores and the accuracy-based collective effect measured by sensitivity such that the relationship between the accuracy-based measure and the component functions can be uncovered.

In order to let the readers follow our SFT analysis steps to replicate our results (e.g., results of C_AND_(*t*), *A*_*AND*_(*t*), and fPCA analysis), please see the Supplementary material (S.1). We upload our script and data into Github. Files can be downloaded at https://github.com/hanekaze/A-new-measure-of-group-decision-making-efficiency.

## Results

Table 2 presents the mean correct RTs and accuracy for all the combinations of the social condition and contrast level.

**Table 2:** Mean correct response time (RT) and accuracy for all the combinations of social condition and contrast level

	25.0%	17.0%	13.5%	11.5%	Average
RT (seconds)					
Worse observer	0.532 (0.056)	0.608 (0.081)	0.692 (0.079)	0.710 (0.097)	0.636 (0.104)
Better observer	0.479 (0.067)	0.528 (0.057)	0.571 (0.058)	0.600 (0.063)	0.545 (0.074)
Collaboration	0.506 (0.052)	0.565 (0.064)	0.629 (0.068)	0.654 (0.070)	0.589 (0.084)
average	0.506 (0.060)	0.567 (0.073)	0.631 (0.082)	0.655 (0.087)	
Accuracy					
Worse observer	0.95 (0.04)	0.81 (0.08)	0.62 (0.07)	0.52 (0.04)	0.72 (0.18)
Better observer	0.97 (0.02)	0.89 (0.04)	0.68 (0.06)	0.56 (0.04)	0.78 (0.17)
Collaboration	0.97 (0.03)	0.87 (0.07)	0.70 (0.07)	0.54 (0.02)	0.77 (0.17)
average	0.96 (0.03)	0.86 (0.07)	0.67 (0.07)	0.54 (0.03)	

Values in parentheses represent standard deviation

### ANOVA

For RT, the results showed a significant main effect of contrast level [*F* (3, 54) = 63.46, *p* < 0.001, ${\eta}_p^2$ = 0.78]. Post hoc comparison showed that the mean RT of the contrast level of 25.0% was the fastest and that the mean RT of the contrast level of 17.0% was faster than that of the contrast level of 13.5% and that of the contrast level of 11.5% (*ps* < .01 for all comparisons). The difference between the contrast levels of 13.5% and 11.5% was not significant. There was a main effect of social condition [*F* (2, 18) = 4.005, *p* = 0.04, ${\eta}_p^2$ = 0.31]. Post hoc comparison showed that the mean RT of the better observer was faster than that of the worse observer (*ps* < .05). However, there were no significant differences between the performance of the collaboration and the better observer or worse observer. The two-way interaction did not reach the significance level.

For accuracy, the results showed that a significant main effect of contrast level [*F* (3, 54) = 560.53, *p* < 0.001, ${\eta}_p^2$ = 0.97]. Post hoc comparison showed that accuracy increased as the contrast level increased and that the differences between every two contrast levels were all significant (*ps* < .01 for all comparisons). Moreover, there was a significant main effect of social condition [*F* (2, 18) = 3.936, *p* = 0.04, ${\eta}_p^2$ = 0.30]. Post hoc comparison showed that the accuracy of the worse observer was lower than that of the better observer (*ps* < .05). However, there were no observable, significant differences between collaboration and the better observer or worse observer. The two-way interaction did not reach the significance level.

### Detection sensitivity

**Fig. 3:**
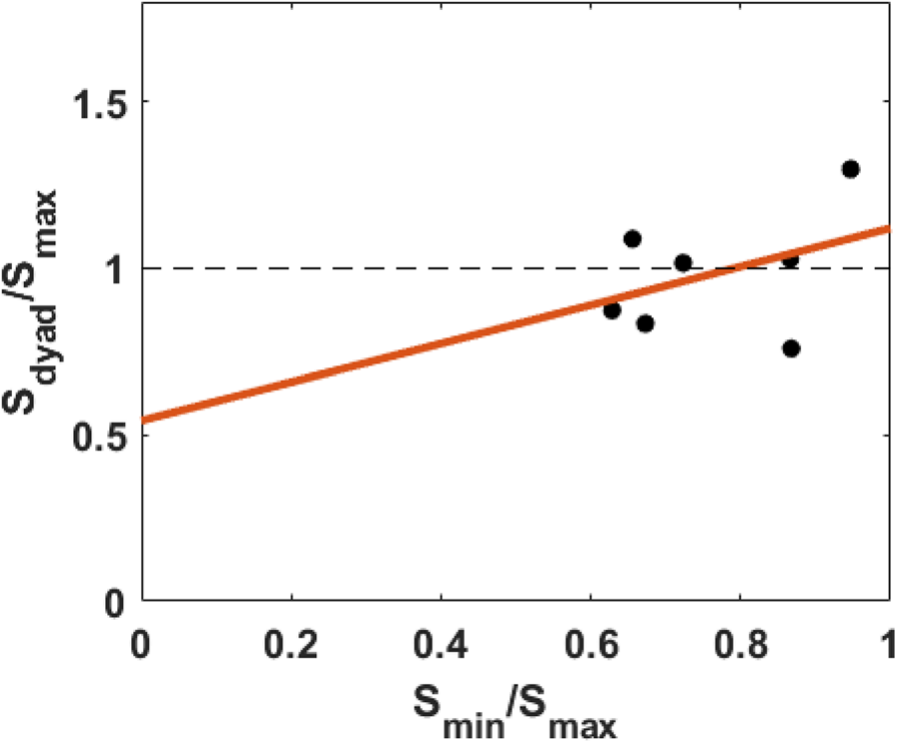
Plot of the accuracy-based collective effect (*Sdyad*/*Smax*) as a function of relative detection sensitivity (*Smin*/*Smax*). The red line represents the regression line

Figure 3 plots the relationship between the relative sensitivity between the worse observer and the better observer and the accuracy-based collective effect (i.e., *S*_*dyad*_/*S*_*max*_). Although we observed a slight trend of positive correlation, as found in the Bahrami et al. (2010) study, this positive correlation did not reach the significance level (*R*^2^ = 0.16, slope = 0.58, *p* = 0.37). The non-significant result may have occurred because of the restriction of range—namely, in the present experiment, the relative detection sensitivities of all dyads were above 0.6.

### Capacity coefficient^8^

**Fig. 4:**
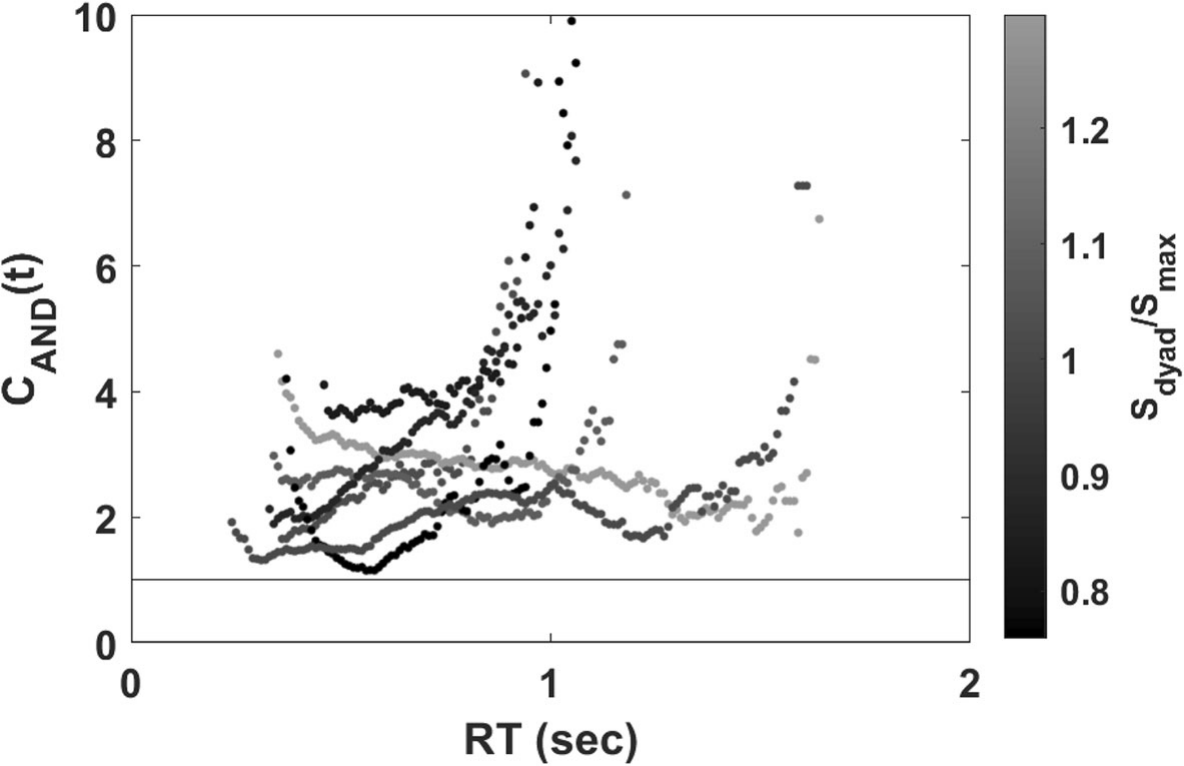
Plot of the capacity coefficient function for each dyad in Experiment 1. The capacity functions were plotted by the level of the accuracy-based collective effect represented by its brightness level

Figure 4 shows the plot of the capacity coefficient function for each dyad. The capacity functions are plotted according to the level of the accuracy-based collective effect (i.e., *S*_*dyad*_/*S*_*max*_) to reveal the relationship between the capacity level and the accuracy-based collective effect. The brightness level represents the level of the accuracy-based collective effect. Our visual inspection indicated that all dyads were of supercapacity with all *C*_*AND*_(*t*) greater than 1 for all times *t*. However, we did not find a robust relationship between the capacity values and the level of the accuracy-based collective effect.

### Assessment function

**Fig. 5:**
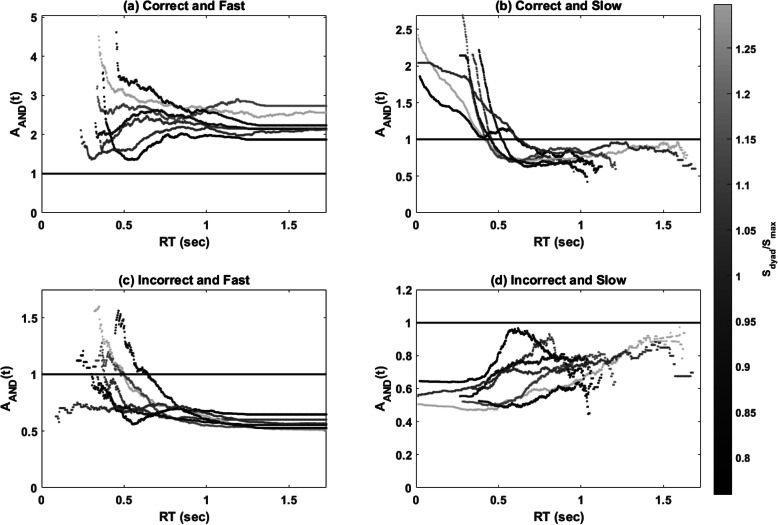
Plots of the assessment function of workload capacity in Experiment 1. The functions were colored by the level of accuracy-based collective effect. **a** correct and fast, **b** correct and slow, **c** incorrect and fast, and **d** incorrect and slow

Figure 5 shows the *A*_*AND*_(*t*) for all four response types; the functions were plotted according to the level of the accuracy-based collective effect. The assessment functions for each response type can be summarized as follows:

1. For the correct and fast responses, values of *A*_*AND*_(*t*) were consistently greater than 1, suggesting that correct collaborative responses were faster and more frequent than expected—that is, supercapacity processing.
2. For the correct and slow responses, values of *A*_*AND*_(*t*) were greater than 1 at the faster RTs and were then less than 1 at the slower RTs. This indicates that correct and slow responses made after time *t* were more probable than expected at faster RTs but less probable than expected at slower RTs.
3. For the incorrect and fast responses, the results showed that most dyads made more incorrect responses by time t at the faster RTs; however, incorrect and fast responses were less probable than expected at the slower RTs.
4. For the incorrect and slow responses, values of *A*_*AND*_(*t*) were less than 1 for all times *t*, indicating that fewer incorrect and slow responses were observed than the expectation from the UCIP model.

To sum up, the results of *A*_*AND*_(*t*) indicate that the collaborative performance was processed more efficiently than the predicted baseline model, suggesting supercapacity processing. However, similar to the results of *C*_*AND*_(*t*), we did not find a strong relationship between *A*_*AND*_(*t*) and the collective effect indicated by relative detection sensitivity. Thus, the current results did not provide evidence in support of the relationship between time-based measures and accuracy-based measures.

### fPCA and quantitative comparison

We employed fPCA with the varimax rotation to decompose *C*_*AND*_(*t*) and *A*_*AND*_(*t*) into several component functions and plotted the factor score of each component against the accuracy-based collective effect to investigate the relationship between the component function and the accuracy-based collective effect. However, we failed to find a robust relationship between the component functions and the accuracy-based collective effect—that is, all the correlations did not reach the significant level (*ps* > .05). Due to the non-significance, we do not present the results in the main text. Please refer to the Supplementary material (S.2) for detailed descriptions of the fPCA results.

## Discussion

In Experiment 1, we conducted a two-interval forced-choice oddball detection task as employed in the study by Bahrami et al. (2010, 2012a, b) but with several modifications on the test procedure. We measured both accuracy and RT, from which we estimated the accuracy-based and time-based collective effects, respectively. Our accuracy results were consistent with the Bahrami et al. (2010) findings. That is, when two individuals had similar detection sensitivity (*S*_*min*_/*S*_*max*_ > 0.8), we observed a collective benefit with *S*_*dyad*_/*S*_*max*_ larger than 1. Note that the sensitivity measure did not reveal a collective benefit in all dyads. In four of seven dyads, *S*_*dyad*_/*S*_*max*_ was larger than 1, while in the other three, the effect was less than or equal to 1.

In addition, the collective benefit was inferred from the RT data in three ways. First, at the mean RT level, we found no significant difference between the mean RT of the collaboration condition and that of the worse or better observer, suggesting a lack of collective benefits at the mean RT level. Second, the observed *C*_*AND*_(*t*) values which were greater than 1 for all times *t*, suggested supercapacity for all dyads—namely, collective benefits were consistently observed for all pairs. Third, when RT and accuracy were combined, the results of *A*_*AND*_(*t*) again supported supercapacity processing. In particular, the results of *A*_*AND*_(*t*) with respect to correct and fast responses showed that dyads made correct and faster responses more frequently than expected, suggesting supercapacity processing. On the other hand, the correct and slower responses were more probable than expected at the faster RTs, also implying supercapacity processing. For the two types of incorrect responses, the results of *A*_*AND*_(*t*) suggested that dyads were more efficient since fewer incorrect responses were made.

It is interesting to observe that, despite the various applied measures that demonstrated the collective effect, the time-based and accuracy-based measures did not show a robust correlation between the two. Here, we came up with two likely explanations for the non-significant correlational results. First, we consider a likely case of the range of restriction. Participants in the present study had a similar detection sensitivity that restricted the full range of possible sensitivities in the general population, which could lead to non-significant sample correlations. To ameliorate a possible restricted range issue in Experiment 2, we introduced a stimulus noise; an extra background white noise was added on top of the visual display of one of the participants, thus heightening the relative differences in detection sensitivity between the two participants. Second, we suspect that there is another reason why the procedure of Experiment 1 was not sensitive enough to measure the time-based collective benefit. In the current setting, participants’ RT was recorded from the onset of a question mark. However, the participants may have already made their decisions while the two intervals were presented; therefore, the RT may reflect only the motor execution time rather than the information accumulation and decision time. To ameliorate this issue and correctly measure the decision time in Experiment 2, we replaced the 2IFC oddball detection task with a yes/no Gabor detection task. With a better estimation of the decision time, we expect to find a robust relationship between the time-based and accuracy-based measures.

### Experiment 2

In Experiment 2, a yes/no Gabor detection task was conducted to enable a better estimation of the information accumulation time and decision time. In addition, we manipulated the transparency of the noise mask that was superimposed on the target stimulus, to manipulate the detection difficulty and thereby estimate the psychometric function. The experiment could be separated into Experiments 2a and 2b depending on whether an additional background noise was introduced to one of the participant’s displays. In Experiment 2a, dyads were tested without any additional background noise such that participants might have had similar detection sensitivity. In Experiment 2b, one of the participant’s displays was covered by additional background noise such that participants might have had different detection sensitivities. This manipulation could solve the problem of the restriction of range raised by Experiment 1 and allowed us to observe the effect of the relative detection sensitivity on collaborative performance. Following the Bahrami et al. (2010) study, we expected that relative detection sensitivity would reduce the collective benefit, which could be observed by both RT- and accuracy-based measures.

## Methods

### Participants

Twenty-six (18 male and eight female; age: 21.2 ± 2.48 years) and 20 (15 male and five female; age: 22.4 ± 1.55 years) undergraduate students at National Cheng Kung University volunteered to participate in Experiments 2a and 2b, respectively. They were randomly assigned and paired into 13 and 10 dyads, respectively. All the participants were right-handed and signed a written informed consent form prior to the experiment. Upon completion of the experiment, each participant received either a total of NTD 140 per hour or class bonus course credits.

### Design, stimuli, and procedure

**Fig. 6:**
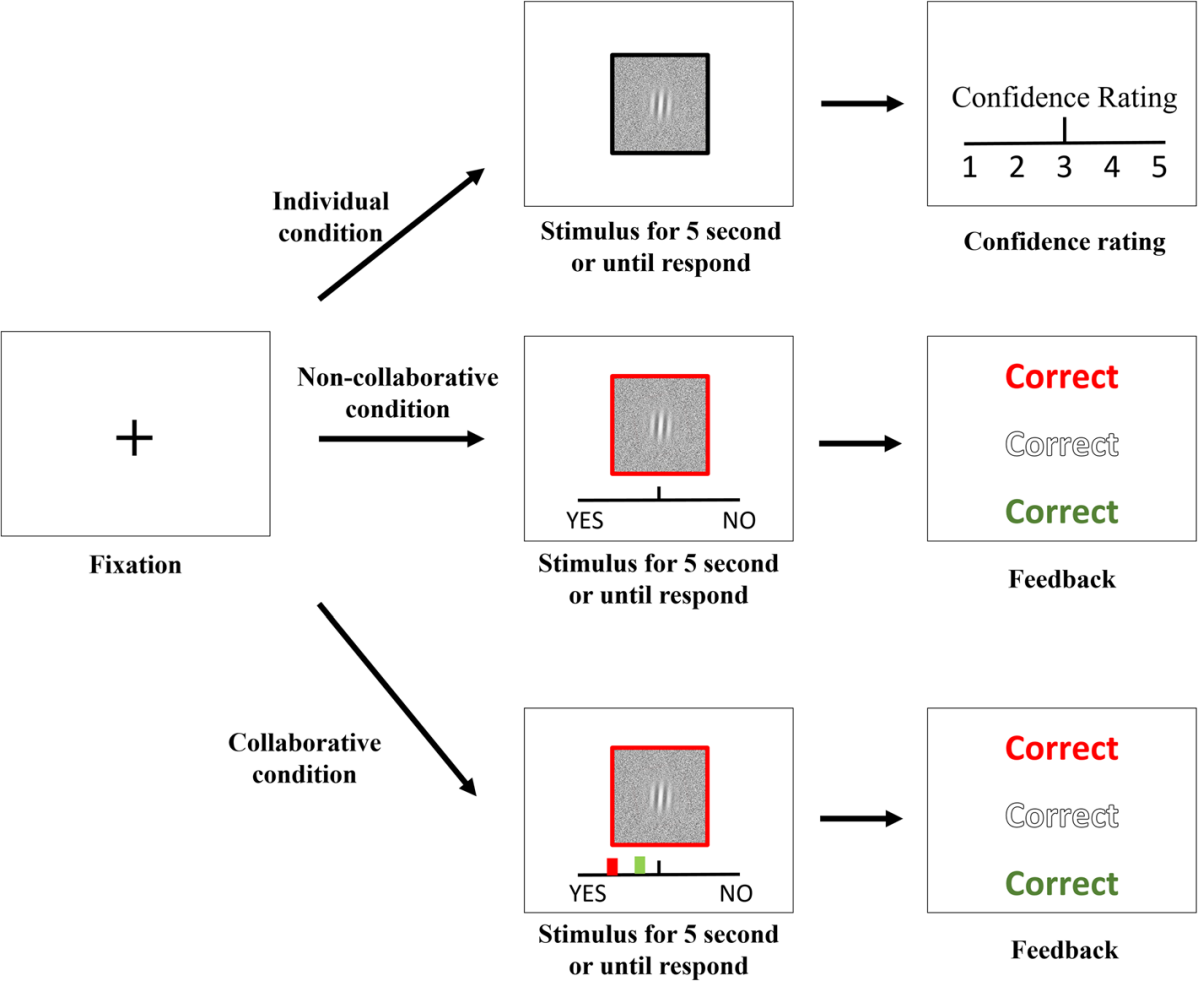
An illustration of the experimental procedure in Experiment 2

The procedure was similar to that used in Experiment 1, though with several modifications. Figure 6 shows an illustration of the trial procedure. The task was to detect the presence of a target as accurately and quickly as possible. The stimulus was presented until a participant delivered a response or 5 s had elapsed. Unlike in Experiment 1, RT had been recorded since the test stimulus was presented. Another difference between Experiments 1 and 2 was that the cue, which informed the participants as to who was going to respond, was the color of the frame of the test stimulus.

**Fig. 7:**
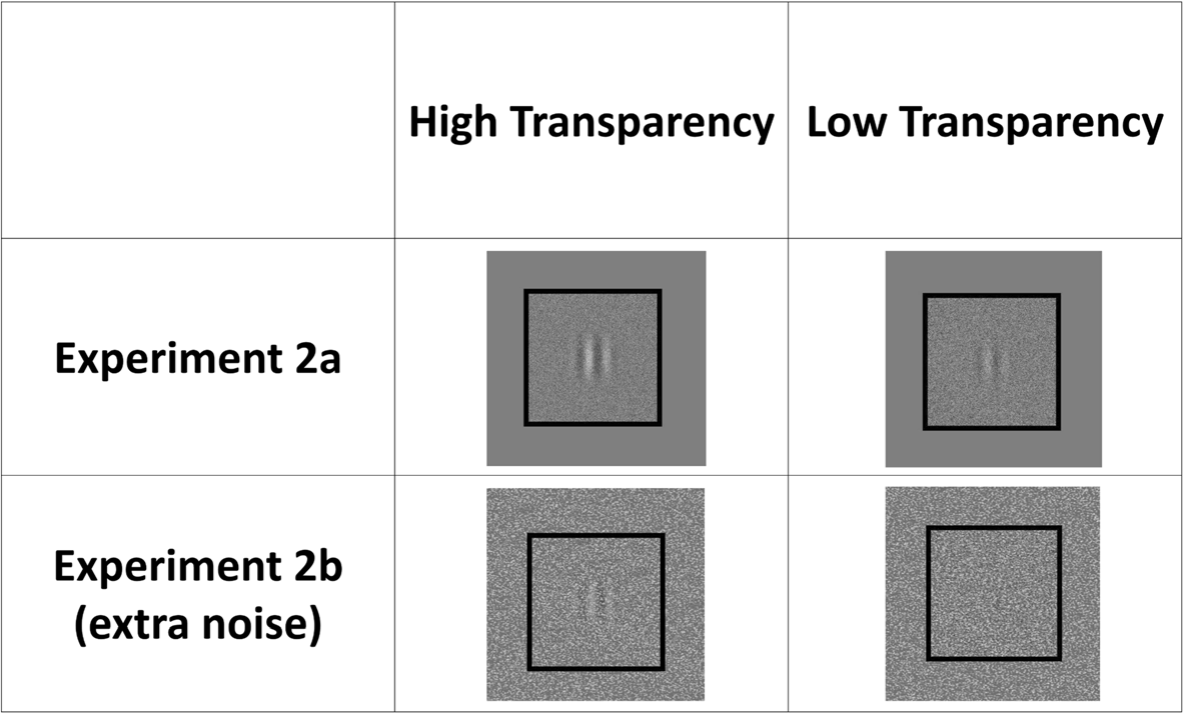
An illustration of stimuli with different levels of noise in Experiment 2

The size of the test stimulus was 256 (horizontal) × 256 (vertical) pixels and the target stimulus was a vertically oriented Gabor patch (standard deviation of the Gaussian envelope: 0.45°; spatial frequency: 1.049 cycles/°; contrast: 10%). In half of the trials, the target was presented and the stimulus was superimposed by a white noise mask with a different degree of transparency to manipulate the detection difficulty. The degree of transparency was adjusted by the value of the alpha channel, i.e., a color component that represents the degree of transparency (or opacity) of a color, with a value ranging from 0 (high transparency) to 1 (low transparency). Hence, if the alpha value was high, it would become difficult to detect the target. Specifically, in Experiment 2a, both participants received the same level of noise, with the alpha value being either 0.81, 0.85, 0.865, 0.885, or 0.9. In Experiment 2b, the alpha value was 0.69, 0.77, 0.83, 0.87, or 0.9. The alpha values were slightly different from those used in Experiment 2a because this adjustment can create a better estimation of the slope of the psychometric function. The critical difference between Experiments 2a and 2b was that extra background noise was introduced into one of the participant’s displays with an alpha value of 0.5. This manipulation would result in relative detection sensitivity—namely, the participant with the extra background noise would have more difficulty detecting the target than would the participant without any extra background noise. Figure 7 shows an example of how we manipulated the transparency of the noise mask and the extra background noise in Experiment 2.

Similar to Experiment 1, participants were required to participate in three cooperation conditions. Each condition was repetitively tested in two sessions in order to collect enough data points. In each session, participants first performed a practice block of 60 trials and then 10 blocks of formal trials. Each block consisted of 2 (presence or absence of the target) × 5 (difficulty levels) × 6 (trials per combination).

### Data analysis

The data analysis procedure was similar to that used in Experiment 1, though with several modifications. For example, for the ANOVA and detection sensitivity analyses, the within-subject factor was the difficulty level, which was manipulated by the alpha value. For the psychometric function analysis, we estimated the probability of responses that the target was presented, denoted as *P*(−*c*), where *c* denotes the alpha value.

## Results

Table 3 presents the mean correct RTs and accuracy for all the combinations of the social conditions and difficulty levels in Experiments 2a and 2b.

**Table 3:** Mean correct response time (RT) and accuracy for all the combinations of social condition and difficulty level (alpha value) in Experiments 2a and 2b

Experiment 2a	0.81	0.85	0.865	0.885	0.9	Average
RT (seconds)						
Worse observer	0.809 (0.097)	0.963 (0.115)	1.049 (0.157)	1.124 (0.159)	1.205 (0.184)	1.030 (0.197)
Better observer	0.753 (0.077)	0.825 (0.081)	0.909 (0.103)	0.978 (0.124)	1.008 (0.159)	0.895 (0.145)
Collaboration	0.782 (0.075)	0.896 (0.089)	0.978 (0.121)	1.054 (0.135)	1.104 (0.165)	0.963 (0.165)
average	0.781 (0.085)	0.895 (0.109)	0.979 (0.138)	1.052 (0.149)	1.106 (0.184)	
Accuracy						
Worse observer	0.91 (0.06)	0.72 (0.13)	0.61 (0.08)	0.46 (0.13)	0.36 (0.19)	0.61 (0.22)
Better observer	0.96 (0.02)	0.85 (0.07)	0.77 (0.07)	0.61 (0.07)	0.48 (0.08)	0.73 (0.18)
Collaboration	0.99 (0.02)	0.95 (0.06)	0.87 (0.12)	0.72 (0.14)	0.57 (0.12)	0.82 (0.18)
average	0.95 (0.05)	0.84 (0.13)	0.75 (0.14)	0.60 (0.16)	0.47 (0.13)	
Experiment 2b	0.69	0.77	0.83	0.87	0.9	average
RT (seconds)						
Worse observer	0.900 (0.222)	0.935 (0.193)	1.082 (0.297)	1.170 (0.300)	1.275 (0.463)	1.072 (0.329)
Better observer	0.748 (0.121)	0.836 (0.189)	1.025 (0.259)	1.165 (0.287)	1.271 (0.470)	1.009 (0.341)
Collaboration	0.823 (0.137)	0.880 (0.147)	1.049 (0.269)	1.170 (0.290)	1.296 (0.451)	1.044 (0.324)
average	0.824 (0.172)	0.884 (0.176)	1.052 (0.267)	1.168 (0.282)	1.281 (0.445)	
Accuracy						
Worse observer	0.89 (0.12)	0.77 (0.16)	0.60 (0.13)	0.48 (0.16)	0.38 (0.18)	0.63 (0.24)
Better observer	0.99 (0.01)	0.97 (0.03)	0.87 (0.11)	0.61 (0.14)	0.37 (0.18)	0.76 (0.27)
Collaboration	0.97 (0.03)	0.92 (0.09)	0.84 (0.12)	0.67 (0.17)	0.45 (0.15)	0.77 (0.22)
average	0.95 (0.08)	0.89 (0.14)	0.77 (0.17)	0.59 (0.17)	0.40 (0.17)	

Values in parentheses represent standard deviation

### ANOVA

#### Experiment 2a

For RT, the results showed a main effect of social condition [*F* (2, 36) = 4.628, *p* = 0.02, ${\eta}_p^2$ = 0.20]. Post hoc comparison showed that the mean RT of a better observer was faster than that of a worse observer (*ps* < .05); however, there was no observable significant difference between collaboration and the worse or better observer. Moreover, the results showed a significant main effect of difficulty level [*F* (4, 144) = 154.23, *p* < 0.001, ${\eta}_p^2$ = 0.81]. Post hoc comparison showed that the mean RT was slower as the difficulty level increased and that the differences between every two difficulty levels were all significant (*ps* < .01 for all comparisons). The two-way interaction did not reach the significance level.

In terms of accuracy, the results showed a significant main effect of the social condition [*F* (2, 36) = 27.59, *p* < 0.001, ${\eta}_p^2$ = 0.61]. Post hoc comparison showed that the accuracy of collaboration was higher than that of the better observer (*ps* < .05) and that of the worse observer (*ps* < .01). Moreover, the accuracy of the better observer was higher than that of the worse observer (*ps* < .01). The results showed a significant main effect of difficulty level [*F* (4, 144) = 345.49, *p* < 0.001, ${\eta}_p^2$ = 0.91]. Post hoc comparison showed that accuracy declined as the difficulty level increased and that the differences between every two difficulty levels were all significant (*ps* < .01 for all comparisons). The interaction effect was also significant [*F* (8, 144) = 4.635, *p* < 0.001, ${\eta}_p^2$ = 0.20]. Post hoc comparison showed that the accuracy of the worse observer was lower than that of the better observer and collaboration when the alpha value was 0.85, 0.865, 0.885, 0.9 (*ps* < .01) but the differences were not significant when the alpha value was 0.81 (i.e., the easiest condition). Moreover, the results showed that the accuracy of collaboration was significantly higher than that of the better observer when the alpha values were 0.885 (*ps* < .01) and 0.85, 0.865, and 0.9 (*ps* < .05), suggesting a collective benefit; however, the difference was not significant when the alpha value was 0.81.

#### Experiment 2b

For RT, the results showed a main effect of difficulty level [*F* (4, 108) = 34.780, *p* < 0.001, ${\eta}_p^2$ = 0.56]. Post hoc comparison showed that the mean RTs were slower when the alpha values were 0.83 and 0.87 than when the alpha values were 0.69 and 0.77 (*ps* < .01). Moreover, the mean RT was slower when the alpha value was 0.9 than when the alpha values were 0.69, 0.77, and 0.83 (*ps* < .01 for all comparisons). No other effects reached the significance level.

In terms of accuracy, the results showed that a main effect of difficulty level [*F* (4, 108) = 121.59, *p* < 0.001, ${\eta}_p^2$ = 0.82]. Post hoc comparison showed that accuracy declined as the difficulty level increased and that the differences between every two difficulty levels were significant (*ps* < .01) except for the difference between the two easiest difficulty levels (i.e., the alpha values were 0.69 and 0.77). Moreover, there was a significant main effect of social condition [*F* (2, 27) = 9.324, *p* < 0.001, ${\eta}_p^2$ = 0.41]. Post hoc comparison showed that the accuracy of the worse observer was lower than that of the better observer and collaboration (*ps* < .01); however, the difference between collaboration and the better observer was not significant. The interaction effect was significant [*F* (8, 108) = 2.763, *p* = 0.008, ${\eta}_p^2$ = 0.17]. Post hoc comparison showed that the accuracy of the worse observer was lower than that of the better observer only when the alpha value was 0.77 or 0.83 (*ps* < .01). Moreover, the accuracy of the worse observer was lower than that of collaboration when the alpha value was 0.77 (*ps* < .05) and 0.83 and 0.87 (*ps* < .01). However, there were no observable differences between collaboration and the better observer.

#### Detection sensitivity

**Fig. 8:**
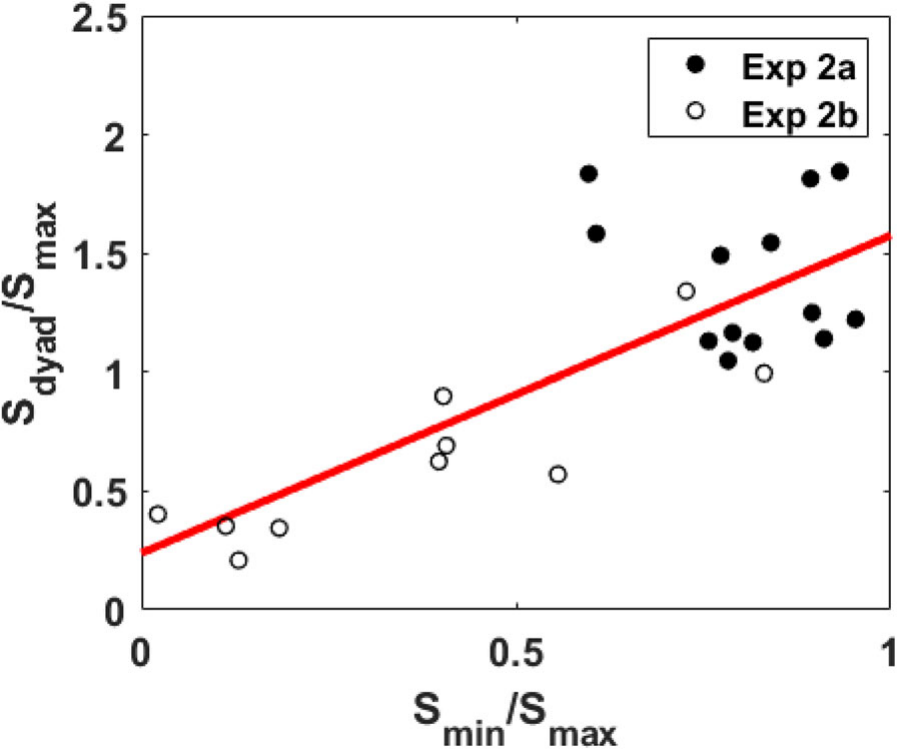
Plot of the accuracy-based collective effect (*Sdyad*/*Smax*) as a function of relative detection sensitivity (*Smin/Smax*). The red line represents the regression line

Figure 8 plots the relationship between the relative detection sensitivity between individuals and the accuracy-based collective effect (i.e., *S*_*dyad*_/*S*_*max*_). The results were similar to what Bahrami et al. (2010) observed and the correlation was significantly positive (*R*^2^ = 0.62, slope = 1.34, *p* < 0.001). Fitted by linear regression, the results suggested that the cutoff for having a collective benefit (*S*_*dyad*_/*S*_*max*_ > 1) was about 0.57.

#### Capacity coefficient

**Fig. 9:**
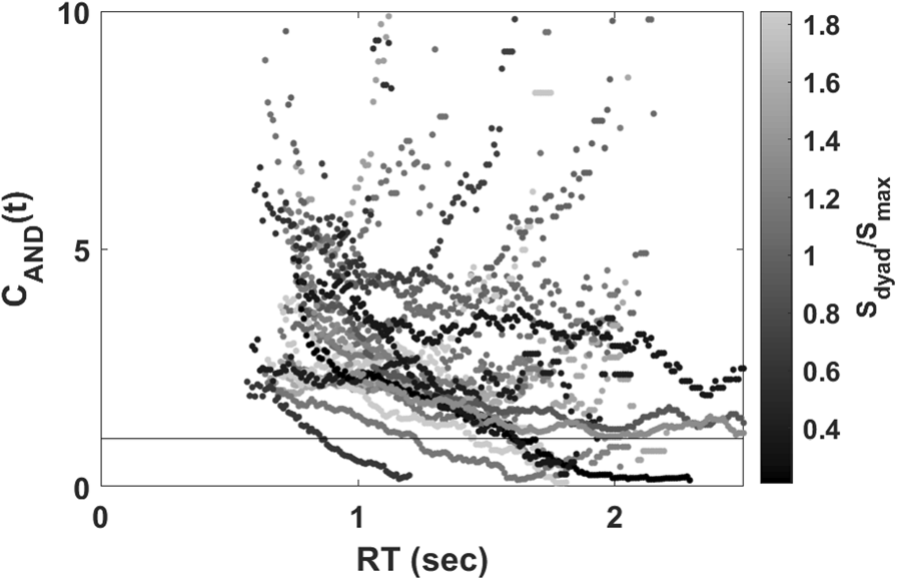
Plot of the capacity coefficient function for each dyad in Experiment 2. The capacity functions were plotted by the level of the accuracy-based collective effect represented by the brightness level

Figure 9 shows the plot of the capacity coefficient function for each dyad. The capacity functions were plotted according to the level of the accuracy-based collective effect to reveal the relationship between the capacity level and the accuracy-based collective effect. The brightness level represents the level of the accuracy-based collective effect. Our visual inspection revealed that most dyads were of supercapacity for all time *t* and that few of them were of limited capacity at the slower RTs. However, similar to Experiment 1, we did not find a robust relationship between the capacity functions (*C*_*AND*_(*t*)) and the level of the accuracy-based collective effect (*S*_*dyad*_/*S*_*max*_).

#### Assessment function

**Fig. 10:**
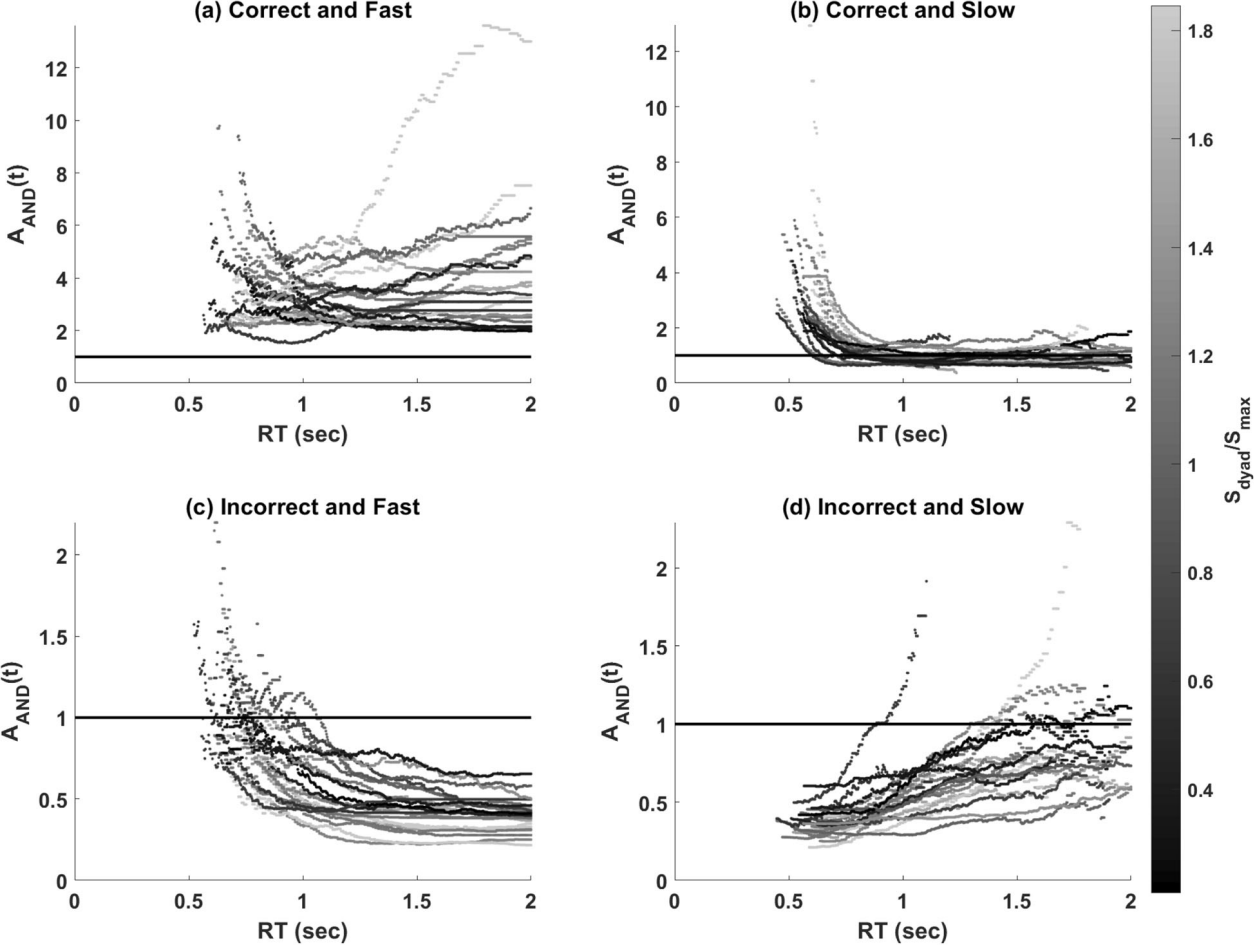
Plots of the assessment function of workload capacity in Experiment 2. The functions were plotted by the level of accuracy-based collective effect. **a** correct and fast, **b** correct and slow, **c** incorrect and fast, and **d** incorrect and slow

Figure 10 shows the *A*_*AND*_(*t*) for all four response types. The functions were plotted according to the level of the accuracy-based collective effect. The assessment functions for each response type can be summarized as follows:

1. For the correct and fast response, values of *A*_*AND*_(*t*) were consistently greater than 1, suggesting that correct group responses were faster and more frequent than expected (i.e., supercapacity processing). Importantly, our visual inspection revealed that *A*_AND_(*t*) systematically increased as a function of the accuracy-based collective effect.
2. For the correct and slow responses, *A*_*AND*_ (*t*) values were above 1 at the faster RTs and reached an asymptote at the value of 1. This suggests that correct and slow responses, made after time *t*, were more probable than expected at the faster RTs.
3. For the incorrect and fast responses, the results showed that most dyads delivered incorrect responses by time *t* more frequently than expected at the faster RTs; however, incorrect and fast responses were less probable than expected at the slower RTs. Moreover, the results suggested that *A*_*AND*_(*t*) values decreased upon an increase in the accuracy-based collective effect.
4. For the incorrect and slow responses, *A*_*AND*_ (*t*) for most dyads was consistently less than 1, indicating that fewer incorrect and slow responses were observed than the expectation from the UCIP model. Similar to the results of the incorrect and fast responses, *A*_*AND*_(*t*) values decreased upon an increase in the accuracy-based collective effect.

To sum up, the pattern of *A*_*AND*_(*t*) was similar to that of Experiment 1, indicating that the group decision-making was of supercapacity. We found that the dyads with a higher accuracy-based collective effect tended to have larger *A*_*AND*_(*t*) values for correct and fast responses and smaller *A*_*AND*_(*t*) values for incorrect responses. Thus, the results converged to suggest the existence of a positive correlation between time-based and accuracy-based measures.

#### fPCA and quantitative comparison

**Fig. 11:**
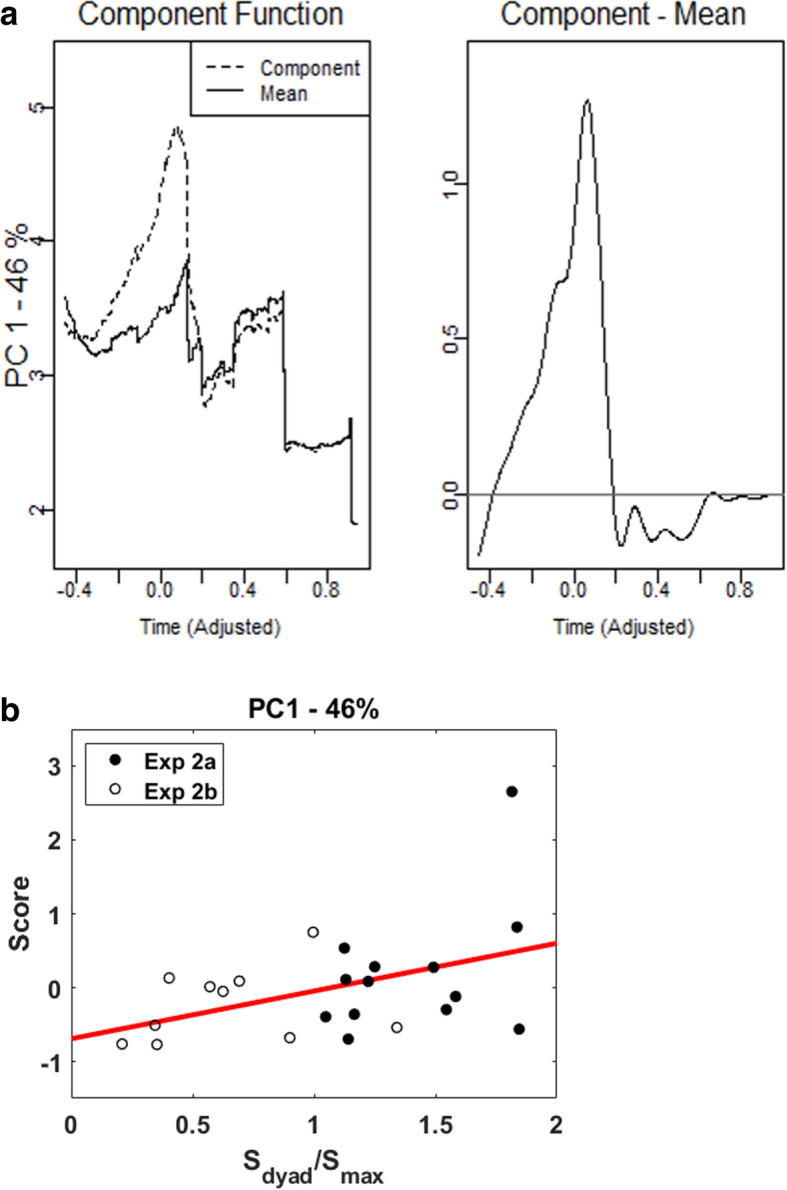
Results of the first principle component of *A(t)* for correct and fast responses. **a** Mean capacity function and the first principal component function (left panel) and the contrast function (right panel). **b** Plot of the time-based measure (the factor score of the first component) against the accuracy-based measure (*Sdyad/Smax*). The red line represents the regression line

When we correlated the factor score of each component with the accuracy-based collective effect (please refer to the Supplementary material (S.3) for detailed results), we found a significant correlation for only the first component of *A*_AND_(*t*) of the correct and fast responses. In Fig. 11a, the left panel shows the capacity function of the first component and the mean capacity function. The right panel shows the contrast function (i.e., the mean capacity function subtracted from the first principal component function). The first principal component function accounts for 46.0% of the variance and indicates a general increase in the capacity values at the faster RTs. In other words, the first component captures the profile of supercapacity processing. Figure 11b shows the scatter plot of the loading of the first component and the accuracy-based collective effect; its correlation reached the significance level (*R*^2^ = 0.19, slope = 0.65, *p* < 0.05).

## Discussion

In Experiment 2, we conducted a yes/no Gabor detection task. Both accuracy and RT were collected to estimate a potential collective benefit. The sensitivity results were consistent with the Bahrami et al. (2010) findings. That is, when the two participants had similar detection sensitivity (i.e., *S*_*min*_/*S*_*max*_ > 0.57), the collective benefit was observed with *S*_*dyad*_/*S*_*max*_ greater than 1. By contrast, when two individuals’ sensitivities were dissimilar (*S*_*min*_/*S*_*max*_ ≤ 0.57), the collective cost was observed.

Similar to Experiment 1, we can infer the collective benefit from the RT data in three ways. First, at the mean RT level, the results were similar to those of Experiment 1, suggesting that there was no collective benefit. Second, the results of *C*_*AND*_(*t*) showed supercapacity with capacity values greater than 1 for all times *t* for most dyads; however, for some dyads, the capacity went from supercapacity to limited capacity as a function of RT. This implied that collective benefit can be found in most dyads but that only a few dyads had collective benefits at the faster RTs. Third, when one combined both RT and accuracy, the results of *A*_*AND*_(*t*) again supported supercapacity processing and showed evidence of the ordered relationship between accuracy-based collective effect and *A*_*AND*_(*t*). In particular, for correct and fast responses, the results of *A*_*AND*_(*t*) showed that dyads made correct and faster responses more frequently than expected, thereby suggesting supercapacity processing. In addition, we observed that the dyads with a higher accuracy-based collective effect showed larger values of *A*_*AND*_(*t*) for the correct and fast responses. On the other hand, for the correct and slow responses, the values of *A*_*AND*_(*t*) were larger than 1 at the faster RTs, also implying supercapacity processing. For the two types of incorrect responses, the results of *A*_*AND*_(*t*) suggested that dyads were of supercapacity because there were fewer incorrect responses than the expectation from the UCIP model.

When we correlated the time-based and accuracy-based measures, we found a significant positive correlation between accuracy-based collective effect and the first principal component of *A*_*AND*_(*t*) of the correct and fast responses. The first principle component indicates an increase in *A*_*AND*_(*t*) for correct and fast responses at the faster RTs, implying that dyads with higher factor scores had a higher level of supercapacity. Hence, the correlation shows that dyads with a higher collective effect in the accuracy-based measure tended to have a larger supercapacity in the time-based measure.

## General discussion

### Summary of the present findings

In the present study, we examined the collective effect in perceptual decision-making tasks, such as a two-interval forced-choice oddball detection task (Experiment l) and a yes/no Gabor detection task (Experiment 2). We followed the Bahrami et al. (2010) study to estimate the accuracy-based collective effect through a comparison of the collaborative detection sensitivity to the sensitivity of the better individual. We utilized SFT to analyze the decision efficiency by comparing the performance of the collaborative condition to the capacity baseline, which assumes that group members work independently in a non-collaborative fashion. We investigated the relationship between the accuracy-based and timed-based measures of the collective effect and proposed that the assessment function of workload capacity *A*_*AND*_(*t*) can be regarded as a novel and diagnostic measure for quantifying group decision-making efficiency.

To address our first goal of replicating the effect of sensitivity similarity on joint decisions, we first conducted the accuracy analyses. The results of Experiment 1 showed that four of the dyads had *S*_*dyad*_/*S*_*max*_ ≥ 1, suggesting collective benefit. The other three dyads had *S*_*dyad*_/*S*_*max*_ < 1, suggesting collective cost. There was a slight trend of a positive correlation between the accuracy-based collective effect and the relative detection sensitivity between observers; however, it did not reach the significance level. The inspection of the measured relative differences in the subjects’ detection sensitivities implied the possibility of a restricted range. The random subject selection had formed dyads of subjects whose detection sensitivities were, overall, more similar than dissimilar. The restriction of the range is a reasonable explanation for the failure to fully replicate the Bahrami et al. (2010) findings. To amend the issue of the restriction of range, Experiment 2 increased the relative differences in detection sensitivity between observers by introducing an extra background noise to one of the participants. In Experiment 2, we found that 15 pairs of participants had *S*_*dyad*_/*S*_*max*_ ≥ 1, suggesting a collective benefit, while the other eight pairs had *S*_*dyad*_/*S*_*max*_ < 1, suggesting a collective cost. It is noted that the cutoff point for observing the collective benefit is when the relative detection sensitivity (*S*_*min*_/*S*_*max*_) equals 0.57. As expected, the effect of inducing more individual variability in detection sensitivities led to the replication of the main findings of Bahrami et al.: a significant correlation between the accuracy-based collective effect and the relative detection sensitivity was observed. This more strongly supported findings of Bahrami et al. that the collective benefit was only observable when the group members had similar levels of detection sensitivity.

We further analyzed RT to address the second goal of testing whether RT and accuracy measures are consistent to draw similar conclusions about the collective effect. We used both *C*_*AND*_(*t*) and *A*_*AND*_(*t*) to quantify the RT-based collective effect. The results of Experiments 1 and 2 were consistent in suggesting that all the pairs had *C*_*AND*_(*t*) and *A*_*AND*_(*t*) of the correct and fast responses larger than 1, implying that they were all of supercapacity in group decision-making—that is, collaborative decision-making is always more efficient than non-collaborative decision-making, although collaboration can have both a benefit and a cost in terms of detection sensitivity (i.e., individual differences in group detection sensitivity). Though we did not observe a robust relationship between *C*_*AND*_(*t*) and the accuracy-based collective effect measured by detection sensitivity (i.e., *S*_*dyad*_/*S*_*max*_), we found an ordered relationship between the accuracy-based collective effect (*S*_*dyad*_/*S*_*max*_) and *A*_*AND*_(*t*) (see Fig. 10). This suggests that both the time-based and accuracy-based measures can capture the collective benefit.

Our last goal is to establish the assessment function of workload capacity as a standard measure of group decision efficiency. It is important to note that while most of the *A*_*AND*_(*t*) analyses and applications are based on the position that *A*_*AND*_(*t*) provides a point estimate of capacity for a single dyad, *A*_*AND*_(*t*) is a function of response time and, as seen in Figs. 5 and 10, its shape changes differently for correct/incorrect and fast/slow response times. One of the recent studies employed an advanced idea to analyze the shape of the *A*_*AND*_(*t*) function by using the fPCA analysis (Burns et al., 2013; Houpt, Blaha, et al., 2014). We also decided to utilize the fPCA analysis on *A*_*AND*_(*t*) to further investigate whether different component functions would provide additional insights into the accuracy measures as well as into the nature of the *A*_*AND*_(*t*) function. Further analysis of the data of Experiment 2 via fPCA showed that it is the first component of *A*_*AND*_(*t*), i.e., an increase of *A*_*AND*_(*t*) values at the faster RTs, which positively correlated with *S*_*dyad*_/*S*_*max*_. That is, the accuracy-based collective benefit may imply a decision-processing advantage (i.e., supercapacity processing and more efficient processing) especially for the faster RTs. The combined fPCA and *A*_*AND*_(*t*) analyses, as a novel and diagnostic tool, offer a potentially promising direction for the study of group decision efficiency, as well as for diagnosing individual differences in terms of how efficiently and accurately group members work together to make a final decision.

To sum up, the current study was motivated primarily by a desire to provide more evidence through which to learn about the underlying processing mechanism of group decision-making. Our study extended the Bahrami et al. (2010) psychophysical method by simultaneously recording the accuracy and RT when participants made group decisions. Following a careful examination of the information accumulation time and decision time in the yes/no Gabor detection task in Experiment 2, several major findings and novel contributions can be noted. First, we replicated Bahrami et al. (2010) main findings, which showed that collaboration benefits detection sensitivity only when group members have similar detection sensitivity. By contrast, collaboration hinders detection sensitivity when individuals’ detection sensitivities are dissimilar. Second, following the SFT approach, both results for group decision efficiency, *C*_*AND*_(*t*) and *A*_*AND*_(*t*), showed that collaborative decision-making is always processed more efficiently than is non-collaborative decision-making, with evidence of supercapacity processing for all pairs throughout Experiments 1 and 2. These results implied that participants were able to utilize their partners’ confidence to boost and improve their decision-making. Third, we found no strong correlation between the accuracy-based collective effect and *C*_*AND*_(*t*), which considers only the processing efficiency of the correct responses. Alternatively, we found a robust relationship between the accuracy-based collective effect and *A*_*AND*_(*t*), which considers both RT and accuracy at the same time. It is notable that the first component of the *A*_*AND*_(*t*) of the correct and fast responses, which captures the signature of an increase in capacity at the faster RTs, is significantly correlated with the accuracy-based collective effect. Based on the converging evidence, we may suggest that the supercapacity processing of the correct and fast trials is closely related to the source of the accuracy-based collective effect, which could imply the sharing of similar mental mechanisms.

### Processing mechanism underlying group decision-making

In the context of group decision-making, supercapacity processing suggests more efficient processing for collaborative decisions in which participants can exchange their confidence than for non-collaborative decisions in which participants work independently without any communication. The supercapacity processing indicates that group performance is better than the UCIP predictions, which assumes that the group decision-making system is an unlimited-capacity, independent, and parallel processing system. The evidence of supercapacity can be used to rule out the statistical facilitation account. This means that the collective benefit is not likely to be an artifact of an increase in the number of individual units.

The results of supercapacity processing may suggest two possible mechanisms. First, it implies that, in a group, all members’ processing efforts, or their individually collected evidence, are accumulated and combined through a series of social interactions. This model for group interaction is equivalent to the cognitive processing model known as coactive processing, which is used to characterize a combination of processing outcomes of several mental structures (Houpt & Townsend, 2011; Schwarz, 1989, 1994; Townsend & Nozawa, 1995). Similarly, as in the coactive processing framework, a collective benefit could arise as the result of a “weighted-and-sum” principle of information integration of the individual contributions. Following this analogy, a “coactive” group member can weigh and integrate their confidence based on their perceptual observation with their partner’s confidence by considering the partner’s credibility based on the previous experience acquired from the trial-by-trial feedback. The details of such a process of combining opinions are beyond the scope of the current study; however, it provides a direction for investigation in future research. Second, supercapacity processing may imply information-sharing between parallel channels (Eidels, Houpt, Altieri, Pei, & Townsend, 2011; Miller, 1982; Mordkoff & Yantis, 1991; Townsend & Wenger, 2004). We were able to observe several types of social interactions while participants worked together on making decisions. In the case of non-verbal communication, in which a participant could see their partner showing confidence in one of the responses, this form of interaction boosted the participant’s accumulation of decision information toward that response. While the current evidence is still insufficient for making strong conclusions about various forms of information exchange between the partners in the tasks, and about whether the present results are due to the coactivation or facilitatory interaction between group members, future studies are encouraged to further explore the difference between the two possibilities.

It is generally accepted that efficient group decision-making may have occurred based on the following considerations: (1) What is the form of social interaction? (i.e., verbal or non-verbal), (2) Is the partner trustworthy or reliable? (i.e., the presence or lack of feedback), and (3) What is the decision input from the partner? (i.e., is the partner’s confidence in the current trial high or low?). First, previous studies have compared the effect of collaboration between verbal communication and non-verbal communication on group detection sensitivity (e.g., Bahrami et al., 2012a). The results showed that both forms of communication can enhance group detection sensitivity through the exchange of decision evidence and decision confidence, respectively. However, in the current context of perceptual decision-making tasks, it is difficult to demonstrate the verbal communication effect on the group decision RT because verbal communication usually takes longer. According to our data, for most trials, it takes less than 2 s to complete a perceptual decision. It seems that such a short time interval is not sufficient for members to communicate with each other verbally. As a result, in our study, we considered only the non-verbal form of collaboration (i.e., internal confidence estimate). Future works are encouraged to modify the tasks or increase the tasks’ difficulty, which could create sufficient time for more verbal communication between group members and, as such, allow this to be explored in more detail. Second, it has been shown that participants can utilize a partner’s confidence to increase group detection sensitivity only when feedback is provided (Bahrami et al., 2010; Sorkin et al., 2001). The reason why the response feedback is important in observing the collective benefit is that a participant can learn whether their partner is trustworthy or reliable. If a partner is consistently making a correct decision, the participant would assign a higher weight to their partner’s confidence. By contrast, if the partner is consistently making an incorrect decision, the participant would assign a lower weight to their confidence. The feedback would help participants adjust the weighting process while integrating the partner’s confidence into the practice of making a group decision. However, to our knowledge, collaborative decision-making efficiency has not been tested without a presentation of the response feedback; therefore, we are still unclear as to whether presenting feedback would, indeed, enhance group decision efficiency as compared to the no-feedback condition. Future studies are encouraged to manipulate the presence/absence of feedback to test its effect on group decision efficiency. Third, if a partner is very evidently confident in their decision, participants may simply follow their partner’s opinions to make a decision or give very high weight to the partner’s confidence. Thus, the decision-making process would become very efficient. On the other hand, if the participant received a low-confidence input, they would need to make a decision by integrating their decision evidence with their partner’s decision confidence, which might slow down the processing for the faster decision-maker. As a result, the efficient group decision may have occurred because the partner’s high-confidence input boosted the decision efficiency. Although we have not yet systematically examined how high/low confidence affects group decision efficiency (due to a limited number of trials), we are open to this possibility and leave it for future study. It is also notable that possibilities (2) and (3) may interactively affect group decision efficiency. That is, when a second partner is very reliable and consistent in making a correct decision, the first partner may simply follow the second partner’s decision to offer a response regardless of whether the confidence is high or low. On the other hand, if the partner is not reliable, the first partner may still need to rely on their perception and integrate it with the second partner’s decision confidence to make a final decision, even in the high-confidence trial. More research with sophisticated analyses would be appropriate to investigate the interaction effects.

### Capacity coefficient and assessment function of workload capacity

It is intriguing to note that, in our study, the accuracy-based measure showed both the benefit and cost of collaboration, whereas the time-based measures (i.e., *C*_*AND*_(*t*) and *A*_*AND*_(*t*)) showed only the benefit of collaboration. A reasonable question to ask is: why are the two measures not tightly connected? If one must choose, which measure should be regarded as being the better one in terms of diagnosing the collective benefit? In the introduction, we mentioned a serious challenge to data interpretation if either measure is considered in isolation: that there may be a time-accuracy tradeoff in making a group decision. That is, a group may make a very fast decision by sacrificing the decision accuracy, or it may make an accurate group decision by slowing down its information accumulation process because social interaction takes time. Therefore, the examination of only one type of measure cannot clearly reveal whether collaboration, indeed, presents an advantage. In a surprising turnaround, we could not find strong evidence for the time-accuracy tradeoff; when we considered both measures at the same time, the correlation between the accuracy-based and time-based collective effects was not strong enough (i.e., there was a correlation only between the accuracy-based collective effect and *A*_*AND*_(*t*)). The absence of evidence of a strong correlation may imply that the two measures are related to different aspects of collective effects. Two possibilities can be considered. First, the baselines for computation and inference of the accuracy-based and time-based collective effects are different. The accuracy-based collective effect is quantified by dividing group detection sensitivity by the sensitivity of the better observer (i.e., *S*_*dyad*_/*S*_*max*_). On the other hand, the time-based collective effect is quantified by comparing group decision efficiency to the UCIP baseline, which is generated from the two individuals’ decision efficiencies when they work independently. Therefore, we would argue that, in the previous condition, the collective benefit is defined only when the performance exceeds the better individual, while, in the latter condition, the collective benefit is defined by the group outperforming the integration of the two individuals’ decision efficiencies. The difference in baselines might explain why both benefits and costs are found in the former condition instead of in the latter condition. The second possibility concerns the cooperation rule for group decisions. For the accuracy-based collective effect, the cooperation rule is not considered. Therefore, it is unclear whether a group decision that outperforms the individual decisions occurred because the group decision follows a “winner-takes-all” rule by making a choice based on the opinion of the more confident member or because the group exhaustively processed the two members’ opinions and made a decision by integrating decision evidence. For the time-based measure, we assessed the AND capacity by assuming that group decisions always take place after all the decision evidence is exhaustively processed. That is, both members’ confidence (i.e., the participant’s own and that of their partner) is always considered when making the final decision. In comparison, Brennan and Enns (2015) used the test of the violation of the race-model inequality; the underlying assumption of the test of the race-model inequality is an OR cooperation rule. However, we believe that the AND rule is a better way to characterize how group members make a coherent decision in the present context of perceptual decision-making. The different assumptions in the decision rules may also explain why we could not find a robust relationship between the accuracy-based and time-based collective effects.

To conclude, we argue that *A*_*AND*_(*t*) could serve as a novel and diagnostic measure of group decision efficiency. *A*_*AND*_(*t*) retains the property of accuracy but can also infer the processing efficiency under both correct and incorrect responses. In addition, *A*_*AND*_(*t*) can provide information about the dynamic changes in processing efficiency as a function of RT. Also, *A*_*AND*_(*t*) can select a specific cooperation rule for the analysis of, and inferences about, group decision efficiency. Therefore, we strongly recommend that researchers test the collective effect by using the *A*_*AND*_(*t*) measure.

## Supplementary information

**Additional file 1.**

## Data Availability

The datasets and codes of the current study are available at https://github.com/hanekaze/A-new-measure-of-group-decision-making-efficiency.

## Footnotes

1 The “wisdom of crowds” does not occur only in a context of working as a group. It can be used to refer to the averaging effect while working in isolation.

2 It is commonly assumed that the first-termination rule is adopted in an OR task, when multiple units are analyzed simultaneously, and in which a decision is determined when either decision unit reaches the decision criterion. However, it should be noted that group decision-making does not necessarily follow the first-termination rule; other forms of decision rules (e.g., majority rule, consensus) may be adopted for group decisions.

3 The coactive models assume the information from multiple decision units is summed into a single unit, and that the sum of information drives the total activation which is then compared to a threshold for decision-making (e.g., Schwarz, 1989, 1994; Townsend & Nozawa, 1995; Houpt & Townsend, 2011).

4 The detection sensitivity is defined as the maximum slope of the psychometric function. With the steeper the slope, the higher the detection sensitivity.

5 In the present study, we used the AND capacity rather than the OR capacity to quantify the group decision efficiency. The AND capacity assesses information-processing efficiency when an exhaustive stopping rule is adopted (i.e., the maximum-time rule) whereas the OR capacity assess the efficiency when a self-terminating rule is adopted (i.e., the minimum-time rule). The reason is that we believe that A group decision is made by reaching the consensus through non-verbal communication and the independent-race model following a winner-takes-all rule is not likely to occur in the current context.

6 It is noted that the current testing procedure is different from that used by Bahrami et al. (2010). In their study, a joint decision was required only when the participants made inconsistent individual decisions. By contrast, in the current setting, all the participants were required to perform the individual condition, non-collaborative condition, and collaborative condition, tested in separate blocks.

7 All the participants did not know each other; thus, the friendship cannot account for the group performance.

8 We conducted a one-way repeated measures analysis of variance (ANOVA) to test the effect of contrast level on the collective effect but the effect did not reach the significance level. Hence, we aggregated all the contrast levels to estimate the capacity coefficient function and the assessment function.
