# Supplementary material for: A new measure of group decision-making efficiency
Source: Cogn Res Princ Implic. 2020 Sep 17;5:45. doi: 10.1186/s41235-020-00244-3 (PMC7498531; doi:10.1186/s41235-020-00244-3)
Supplement: Supplementary file 1 — Additional file 1. [file 41235_2020_244_MOESM1_ESM.docx]

Supplementary Material (S.1)

*Implementation*

In this section, we introduce a tutorial using the data of the present study for the implementation of the SFT methodology on the analyses of $C_{AND}(t)$, $A_{AND}(t)$, and fPCA. The data should be organized as the example data and please see Table S.1.1 for the descriptions about each variable. Please see the following for step-by-step instructions. More details including the Matlab and R codes can be found at the Github (https://github.com/hanekaze/A-new-measure-of-group-decision-making-efficiency).

*Table S.1.1 Introduction to each variable.*

| *Variable* | Description |
| --- | --- |
| *Subject* | Group ID |
| *Correct* | A logical value indicating whether the response is correct or incorrect |
| *RT* | A numeric value indicating the RT on a given trial |
| *Channel1* | A logical value indicating whether the participant A is involved in decision making on a given trial |
| *Channel2* | A logical value indicating whether the participant B is involved in decision making on a given trial |

*Capacity coefficient*

The files including capacity_exp1.m, capacity_exp2.m, and edf.m should be arranged in the same folder. After running the capacity_exp1.m or capacity_exp2.m, the capacity coefficient can be computed, and the corresponding figures are generated.

How it works:

1. The data (exp1 collaborate.xlsx and exp1 noncollaborate.xlsx for Experiment 1; exp2a collaborate.xlsx, exp2a noncollaborate.xlsx, exp2b collaborate.xlsx and exp2b noncollaborate.xlsx for Experiment 2) is first loaded. Correct RTs within a range of the 2.5% quartile and the 97.5% quartile are extracted for the analyses.

2. The empirical distribution function for each condition is calculated by using the function edf.m.

t=[0:0.01:5];

y=RTdata;

F=edf(t,y);

where *F* denotes the empirical distribution function, *t* denotes the time span, and *y* denotes the RT data.

3. The capacity coefficient is estimated and plotted.

ct = (log(F_1.*F_2))./(log(F_12));

ct(ct==0)=nan;

plot (t,ct);

where ct denotes $C_{AND}\left( t \right)$.

*Assessment function of workload capacity*

Files including assessment_exp1.R, assessment_Exp1_plot.m, assessment_exp2.R, and assessment_Exp2_plot.m should be arranged in the same folder. After running assessment_exp1.R or assessment_exp2.R, the assessment function can be computed, and the results are saved in the excel files. In addition, Matlab programs (Exp1_plot.m or assessment_Exp2_plot.m) are used to generate the figures.

How assessment_exp1.R or assessment_exp2.R works:

1. The data (exp1 collaborate.xlsx and exp1 noncollaborate.xlsx for Experiment 1; exp2a collaborate.xlsx, exp2a noncollaborate.xlsx, exp2b collaborate.xlsx, and exp2b noncollaborate.xlsx for Experiment 2) is first loaded. Correct RTs within a range of the 2.5% quartile and the 97.5% quartile are extracted for the analyses.

2. The assessment function is computed by the function assessmentGroup. For more details, please see Houpt et al. (2014).

a.and.if <- assessmentGroup(data, stopping.rule = “AND”, correct=FALSE, fast=TRUE, plotAt=T)

3. The results are then saved in the excel file.

Tmp <- rbind(matrix(a.and.if$time,nrow = 1),a.and.if$At.fn)

write.csv(tmp, "Exp1 and if.csv",na = "NaN")

*fPCA*

Files including fPCA_Exp1.R and fPCA_Exp2.R should be arranged in the same folder. The fPCA analyses are conducted by running fPCA_Exp1.R or fPCA_Exp2.R. In addition, the results are saved in the text files.

How it works:

1. The data is the same as used for the analysis of assessment function.

2. The fPCA results are computed by the function fPCAassessment.

a.and.cf <- fPCAassessment(data, 2, stopping.rule= “AND”, correct=TRUE, fast=TRUE, register=c("median"), plotPCs=T)

where the second variable denotes the number of principal functions; here, the number of principal functions is set as 2.

3. The results are saved in the text files.

sink('fPCA_At_cf_Exp2.txt')

a.and.cf

sink()

Supplementary Material (S.2)

*fPCA results of Experiment 1*

*Capacity coefficient*

Figure S.2.1a shows the amount of variance before varimax rotation that can be explained as a function of the number of eigenfunctions. The results suggested a two-components solution. Figure S.2.1b shows the mean capacity function and the principal component function (left panel) and the contrast function (right panel)*.* The first principal component function accounts for 55% of the variance and indicates a general increase of the capacity coefficient at the slower RTs. The correlation between the loading of the first component and the accuracy-based collective effect did not reach the significance level ($R^{2}$ = 0.19, slope = -1.17, *p* = 0.33) (Figure S.2.1c). The second principal component function explains 42% of the variance and indicates an increase in capacity at the faster RTs. The correlation between the loading of the second component and the accuracy-based collective effect did not reach the significance level ($R^{2}$ = 0.18, slope = 1.01, *p* = 0.34) (Figure S.2.1d).

*Assessment function for correct and fast responses*

Figure S.2.2a shows the amount of variance before varimax rotation that can be explained as a function of the number of eigenfunctions. The results suggested a two-components solution. Figure S.2.2b shows the mean assessment function for correct and fast responses and the principal component function (left panel) and the contrast function (right panel)*.* The first principal component function accounts for 81% of the variance and indicates a general increase of the capacity coefficient at the faster RTs. The correlation between the loading of the first component and the accuracy-based collective effect did not reach the significance level ($R^{2}$ = 0.18, slope = 0.51, *p* = 0.35) (Figure S.2.2c). The second principal component function explains 16% of the variance and indicates an increase in capacity at the slower RTs. The correlation between the loading of the second component and the accuracy-based collective effect did not reach the significance level ($R^{2}$ = 0.13, slope = 0.20, *p* = 0.43) (Figure S.2.2d).

*Assessment function for correct and slow responses*

Figure S.2.3a shows the amount of variance before varimax rotation that can be explained as a function of the number of eigenfunctions. The results suggested a three-components solution. Figure S.2.3b shows the mean assessment function for correct and slow responses and the principal component function (left panel) and the contrast function (right panel). The first principal component function accounts for 56% of the variance and indicates a general increase of the capacity coefficient at the faster RTs. The correlation between the loading of the first component and the accuracy-based collective effect did not reach the significance level ($R^{2}$ = 0.15, slope = -0.09, *p* = 0.38) (Figure S.2.3c). The second principal component function explains 33% of the variance and indicates an increase in capacity at the intermediate time window. The correlation between the loading of the second component and the accuracy-based collective effect did not reach the significance level ($R^{2}$ = 0.04, slope = 0.03, *p* = 0.68) (Figure S.2.3d). The third principal component function explains 9% of the variance and indicates an increase in capacity at the slower RTs. The correlation between the loading of the third component and the accuracy-based collective effect did not reach the significance level ($R^{2}$ = 0.05, slope = -0.02, *p* = 0.63) (Figure S.2.3e).

*Assessment function for incorrect and fast responses*

Figure S.2.4a shows the amount of variance before varimax rotation that can be explained as a function of the number of eigenfunctions. The results suggested a two-components solution. Figure S.2.4b shows the mean assessment function for incorrect and fast responses and the principal component function (left panel) and the contrast function (right panel). The first principal component function accounts for 84% of the variance and indicates a general increase of the capacity coefficient at the faster RTs. The correlation between the loading of the first component and the accuracy-based collective effect did not reach the significance level ($R^{2}$ = 0.07, slope = 0.07, *p* = 0.55) (Figure S.2.4c). The second principal component function explains 14% of the variance and indicates an increase in capacity at the lower RTs. The correlation between the loading of the second component and the accuracy-based collective effect did not reach the significance level ($R^{2}$ = 0.08, slope = 0.03, *p* = 0.54) (Figure S.2.4d).

*Assessment function for incorrect and slow responses*

Figure S.2.5a shows the amount of variance before varimax rotation that can be explained as a function of the number of eigenfunctions. The results suggested a two-components solution. Figure S.2.5b shows the mean assessment function for incorrect and slow responses and the principal component function (left panel) and the contrast function (right panel). The first principal component function accounts for 80% of the variance and indicates a general increase of the capacity coefficient at the faster RTs. The correlation between the loading of the first component and the accuracy-based collective effect did not reach the significance level ($R^{2}$ = 0.29, slope = -0.22, *p* = 0.21) (Figure S.2.5c). The second principal component function explains 17% of the variance and indicates an increase in capacity at the slower RTs. The correlation between the loading of the second component and the accuracy-based collective effect did not reach the significance level ($R^{2}$ = 0.08, slope = -0.05, *p* = 0.53) (Figure S.2.5d).

| *(a)* 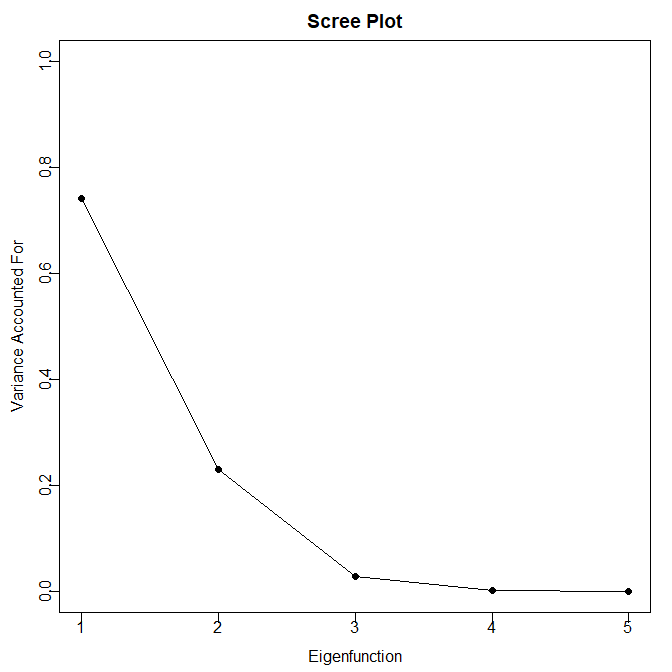 |
| --- |
| *(b)* 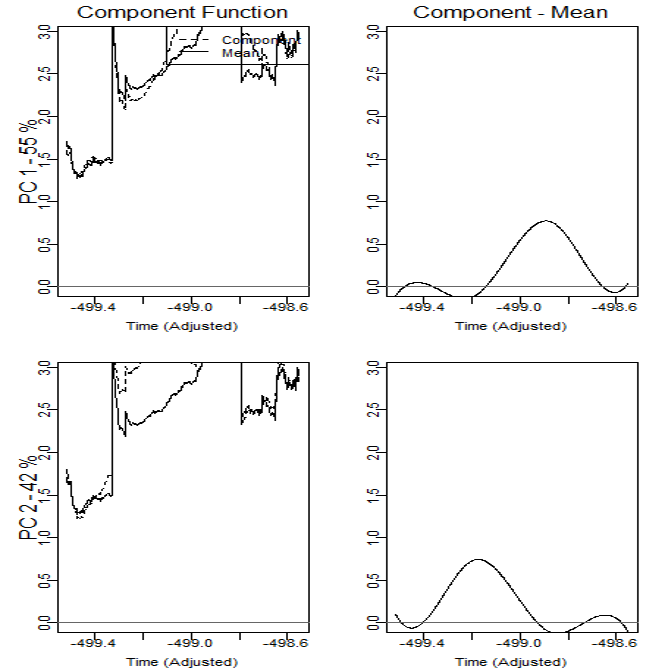 |
| *(c)* 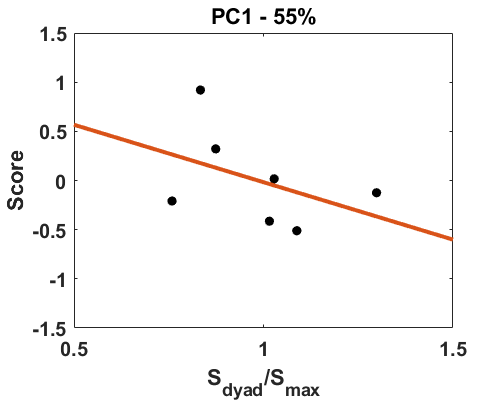 |
| *(d)* 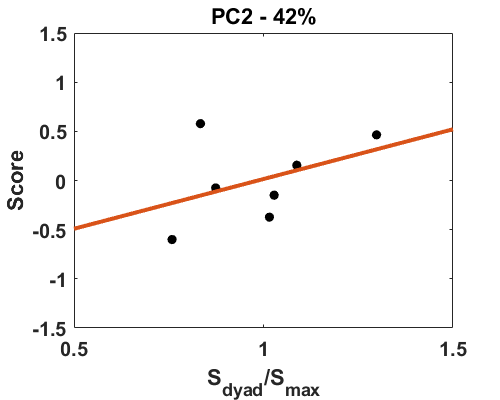 |

*Figure S.2.1*. fPCA of capacity coefficient function. (a) A scree plot showing the amount of variance before varimax rotation accounted for by each eigenfunction, ordered from highest to lowest. (b) Mean capacity function and the principal component function (left panel) and the contrast function (right panel). (c) Plot of the time-based measure (factor score of the first component) against accuracy-based measure (Sdyad/Smax). (d) Plot of the time-based measure (factor score of the second component) against accuracy-based measure (Sdyad/Smax).

| *(a)* 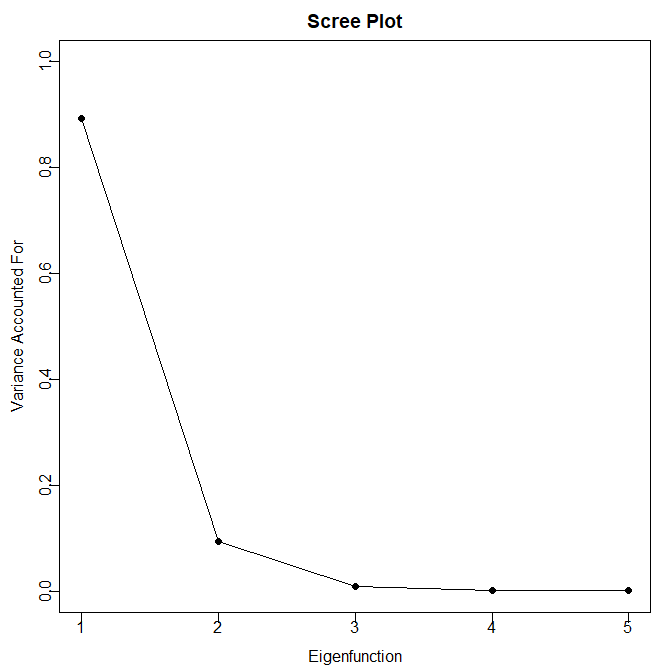 |
| --- |
| *(b)*  *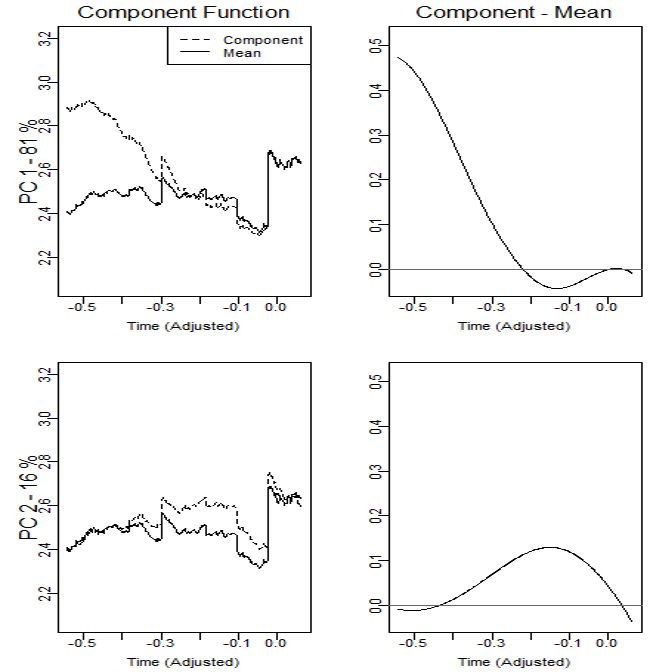* |
| *(c)*  *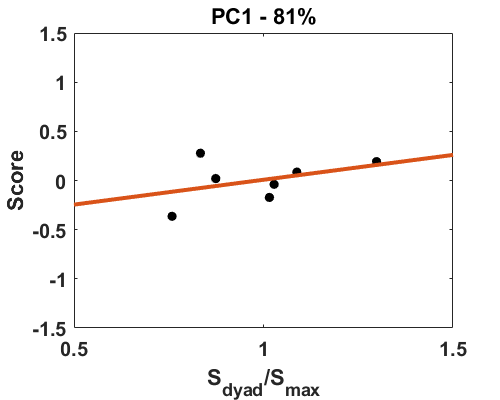* |
| *(d)*  *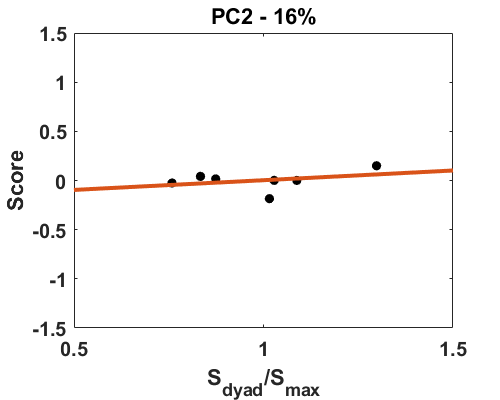* |

*Figure S.2.2*. fPCA of assessment function for correct and fast responses. (a) A scree plot showing the amount of variance before varimax rotation accounted for by each eigenfunction, ordered from highest to lowest. (b) Mean assessment function for correct and fast responses and the principal component function (left panel) and the contrast function (right panel). (c) Plot of the time-based measure (factor score of first component) against accuracy-based measure (Sdyad/Smax). (d) Plot of the time-based measure (factor score of second component) against accuracy-based measure (Sdyad/Smax).

| *(a)* 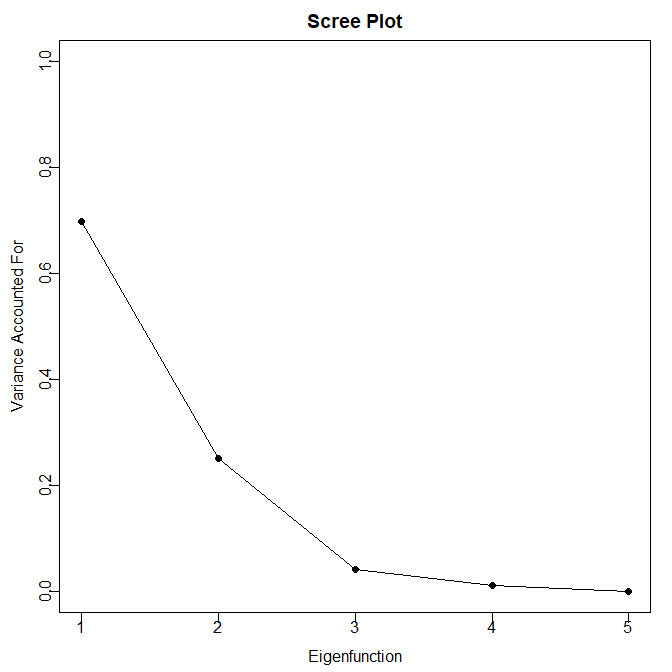 |
| --- |
| *(b)* 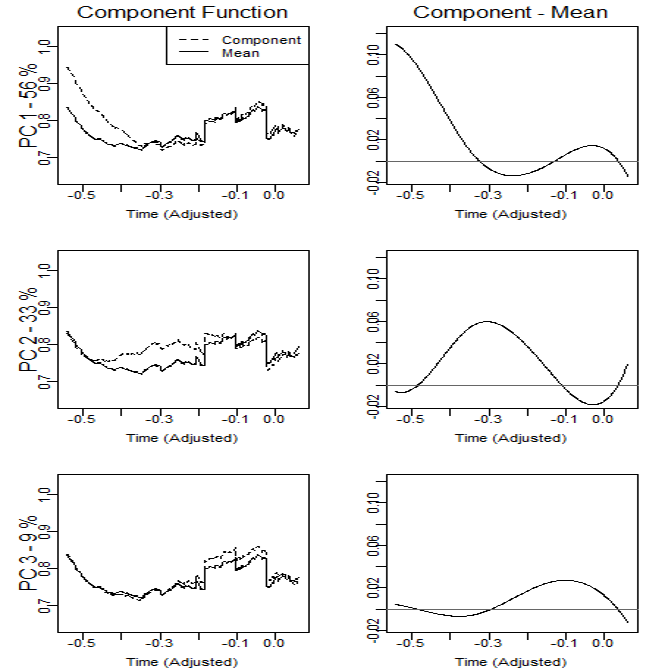 |
| *(c)* 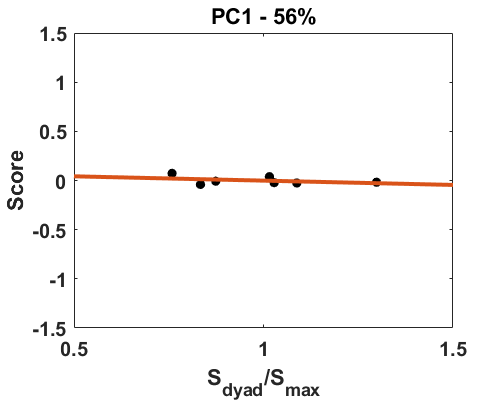 |
| *(d)*  *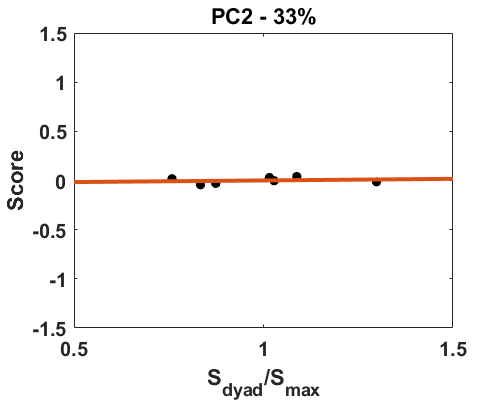* |
| *(e)* 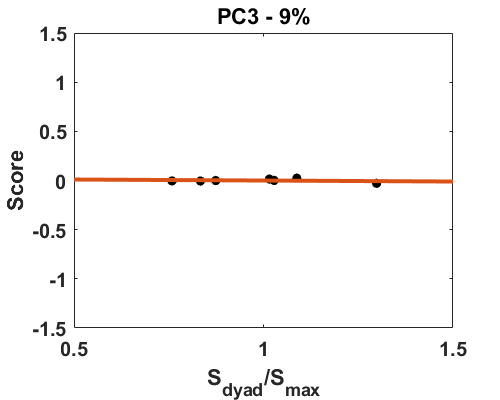 |

*Figure S.2.3*. fPCA of assessment function for correct and slow responses. (a) A scree plot showing the amount of variance before varimax rotation accounted for by each eigenfunction, ordered from highest to lowest. (b) Mean assessment function for correct and slow responses and the principal component function (left panel) and the contrast function (right panel). (c) Plot of the time-based measure (factor score of the first component) against accuracy-based measure (Sdyad/Smax). (d) Plot of the time-based measure (factor score of the second component) against accuracy-based measure (Sdyad/Smax). (e) Plot of the time-based measure (factor score of the third component) against accuracy-based measure (Sdyad/Smax).

| *(a)* 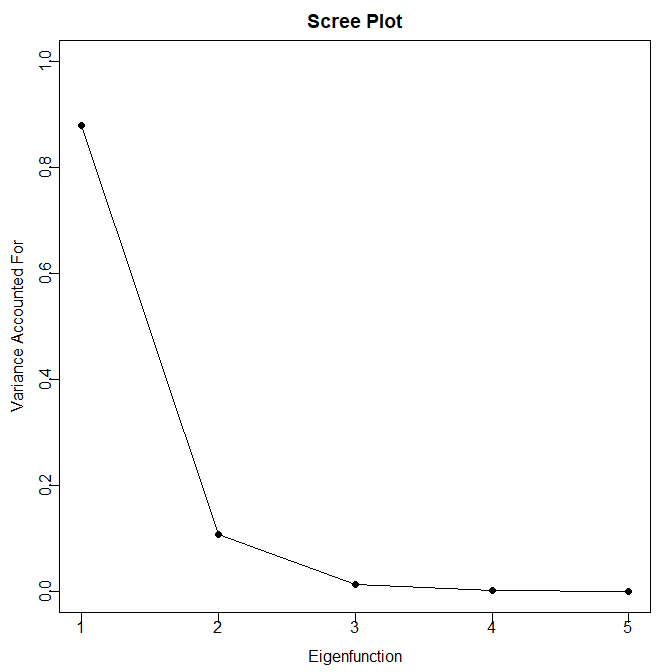 |
| --- |
| *(b)* 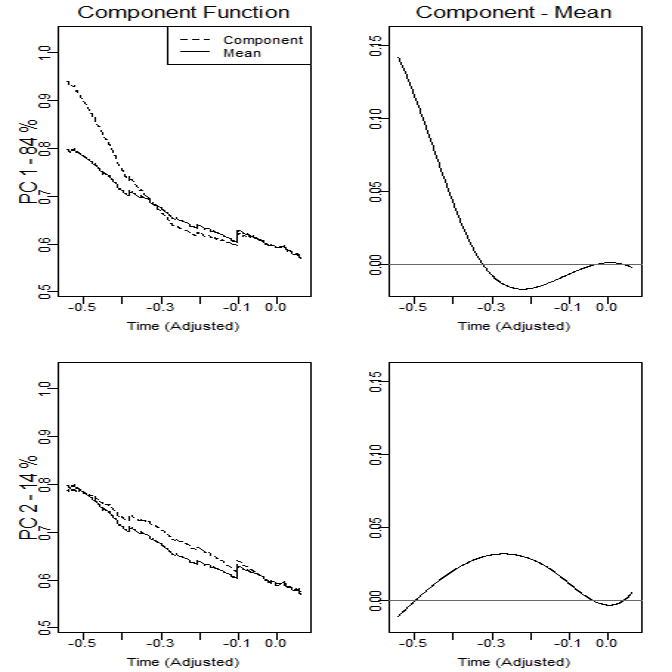 |
| *(c)* 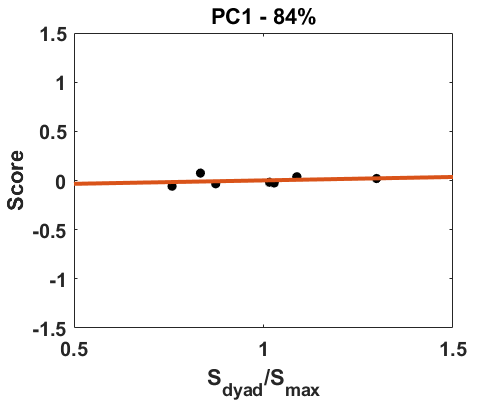 |
| *(d)* 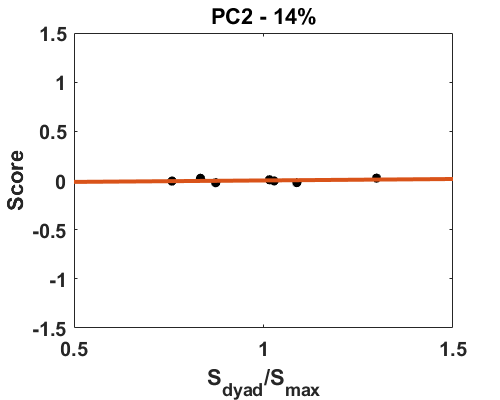 |

*Figure S.2.4*. fPCA of assessment function for incorrect and fast responses. (a) A scree plot showing the amount of variance before varimax rotation accounted for by each eigenfunction, ordered from highest to lowest. (b) Mean assessment function for incorrect and fast responses and the principal component function (left panel) and the contrast function (right panel). (c) Plot of the time-based measure (factor score of the first component) against accuracy-based measure (Sdyad/Smax). (d) Plot of the time-based measure (factor score of the second component) against accuracy-based measure (Sdyad/Smax).

| *(a)* 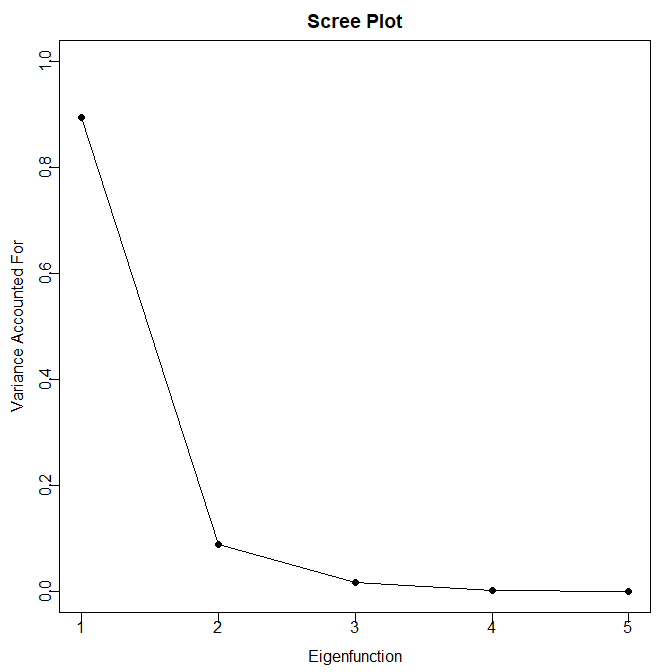 |
| --- |
| *(b)* 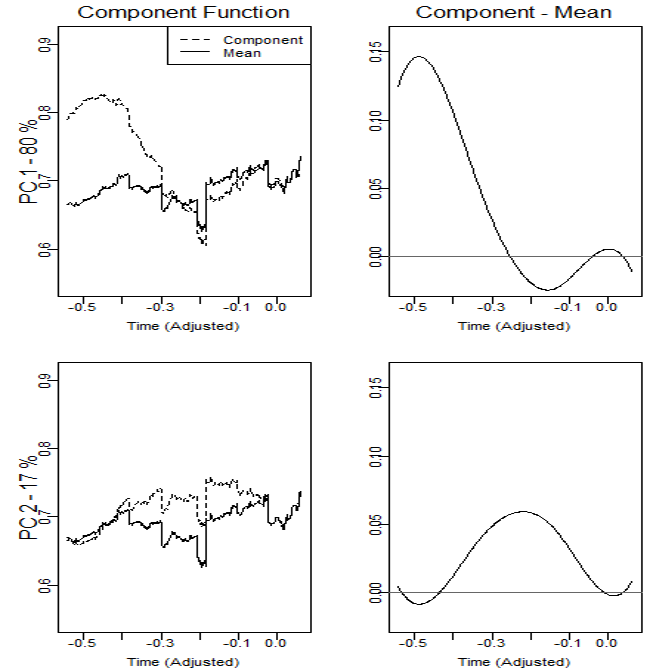 |
| *(c)* 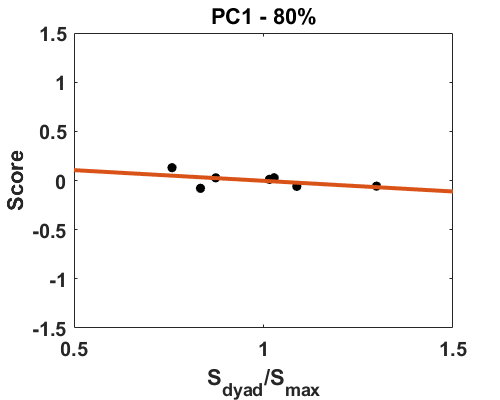 |
| *(d)* 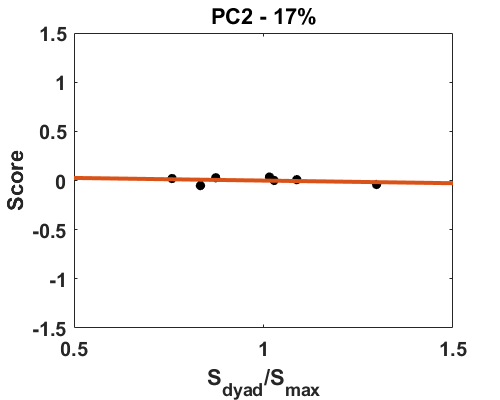 |

*Figure S.2.5*. fPCA of assessment function for incorrect and slow responses. (a) A scree plot showing the amount of variance before varimax rotation accounted for by each eigenfunction, ordered from highest to lowest. (b) Mean assessment function for incorrect and slow responses and the principal component function (left panel) and the contrast function (right panel). (c) Plot of the time-based measure (factor score of the first component) against accuracy-based measure (Sdyad/Smax). (d) Plot of the time-based measure (factor score of the second component) against accuracy-based measure (Sdyad/Smax).

Supplementary Material (S.3)

*fPCA results of Experiment 2*

*Capacity coefficient*

Figure S.3.1a shows the amount of variance before varimax rotation that can be explained as a function of the number of eigenfunctions. The results suggested a two-components solution. Figure S.3.1b shows the mean capacity function and the principal component function (left panel) and the contrast function (right panel)*.* The first principal component function accounts for 46% of the variance and indicates a general increase of the capacity coefficient at the faster RTs. The correlation between the loading of the first component and the accuracy-based collective effect did not reach the significance level ($R^{2}$ = 0.02, slope = -0.34, *p* = 0.49) (Figure S.3.1c). The second principal component function explains 26% of the variance and indicates an increase in capacity at the slower RTs. The correlation between the loading of the second component and the accuracy-based collective effect did not reach the significance level ($R^{2}$ = 0.03, slope = -0.28, *p* = 0.46) (Figure S.3.1d).

*Assessment function for correct and fast responses*

Figure S.3.2a shows the amount of variance before varimax rotation that can be explained as a function of the number of eigenfunctions. The results suggested a two-components solution. Figure S.3.2b shows the mean assessment function for correct and fast responses and the principal component function (left panel) and the contrast function (right panel)*.* The first principal component function accounts for 46% of the variance and indicates a general increase of the capacity coefficient at the faster RTs. The correlation between the loading of the first component and the accuracy-based collective effect as significant ($R^{2}$ = 0.19, slope = 0.65, *p* = 0.04) (Figure S.3.2c). The second principal component function explains 37% of the variance and indicates an increase in capacity at the very fast and slower RTs. The correlation between the loading of the second component and the accuracy-based collective effect did not reach the significance level ($R^{2}$ = 0.04, slope = 0.27, *p* = 0.36) (Figure S.3.2d).

*Assessment function for correct and slow responses*

Figure S.3.3a shows the amount of variance before varimax rotation that can be explained as a function of the number of eigenfunctions. The results suggested a two-components solution. Figure S.3.3b shows the mean assessment function for correct and slow responses and the principal component function (left panel) and the contrast function (right panel)*.* The first principal component function accounts for 63% of the variance and indicates a general increase of the capacity coefficient at the faster RTs. The correlation between the loading of the first component and the accuracy-based collective effect did not reach the significance level ($R^{2}$ = 0.06, slope = 0.07, *p* = 0.26) (Figure S.3.3c). The second principal component function explains 20% of the variance and indicates an increase in capacity at the slower RTs. The correlation between the loading of the second component and the accuracy-based collective effect did not reach the significance level ($R^{2}$ = 0.00, slope = 0.00, *p* = 0.95) (Figure S.3.3d).

*Assessment function for incorrect and fast responses*

Figure S.3.4a shows the amount of variance before varimax rotation that can be explained as a function of the number of eigenfunctions. The results suggested a two-components solution. Figure S.3.4b shows the mean assessment function for incorrect and fast responses and the principal component function (left panel) and the contrast function (right panel)*.* The first principal component function accounts for 70% of the variance and indicates a general increase of the capacity at the faster RTs. The correlation between the loading of the first component and the accuracy-based collective effect did not reach the significance level ($R^{2}$ = 0.15, slope = -0.09, *p* = 0.07) (Figure S.3.4c). The second principal component function explains 20% of the variance and indicates a decrease in capacity at the faster times. The correlation between the loading of the second component and the accuracy-based collective effect did not reach the significance level ($R^{2}$ = 0.06, slope = 0.03, *p* = 0.25) (Figure S.3.4d).

*Assessment function for incorrect and slow responses*

Figure S.3.5a shows the amount of variance before varimax rotation that can be explained as a function of the number of eigenfunctions. The results suggested a two-components solution. Figure S.3.5b shows the mean assessment function for incorrect and slow responses and the principal component function (left panel) and the contrast function (right panel). The first principal component function accounts for 46% of the variance and indicates a general increase of the capacity coefficient at the faster RTs. The correlation between the loading of the first component and the accuracy-based collective effect did not reach the significance level ($R^{2}$ = 0.02, slope = -0.03, *p* = 0.49) (Figure S.3.5c). The second principal component function explains 37% of the variance and indicates an increase in capacity at the very fast and slower RTs. The correlation between the loading of the second component and the accuracy-based collective effect did not reach the significance level ($R^{2}$ = 0.16, slope = -0.08, *p* = 0.06) (Figure S.3.5d).

| *(a)* 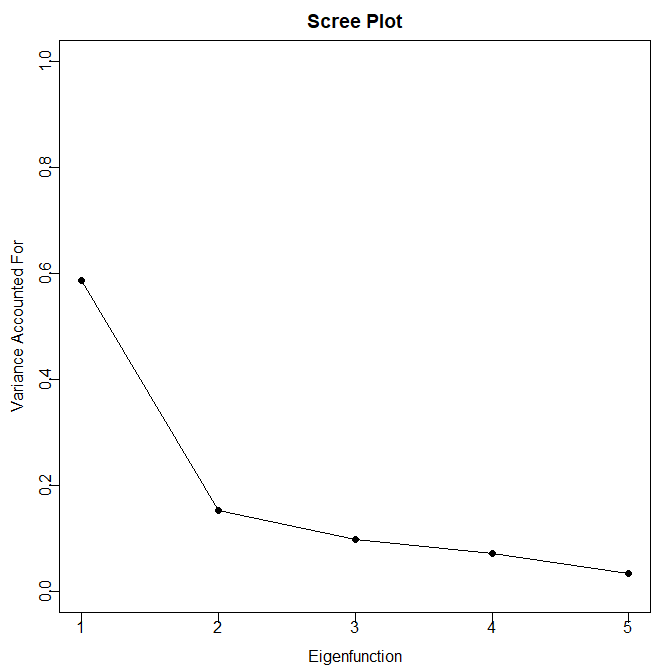 |
| --- |
| *(b)* 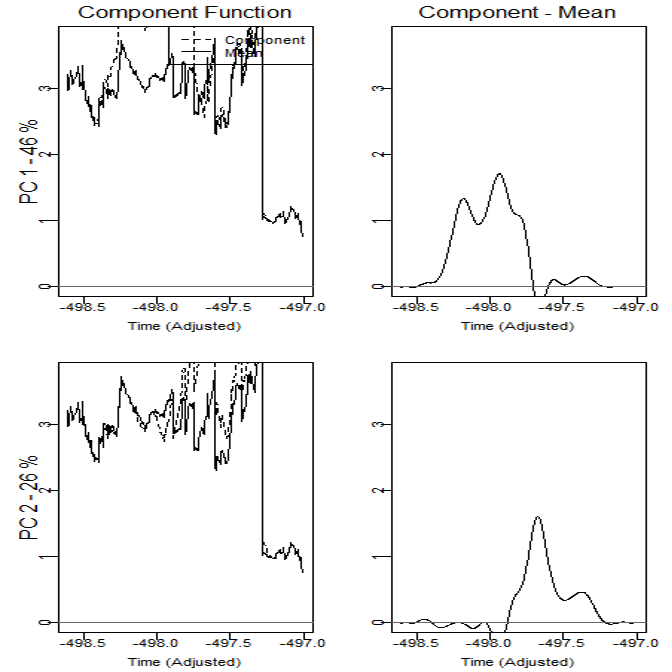 |
| *(c)*  *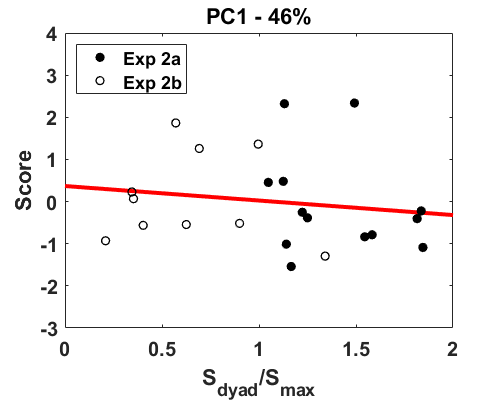* |
| *(d)*  *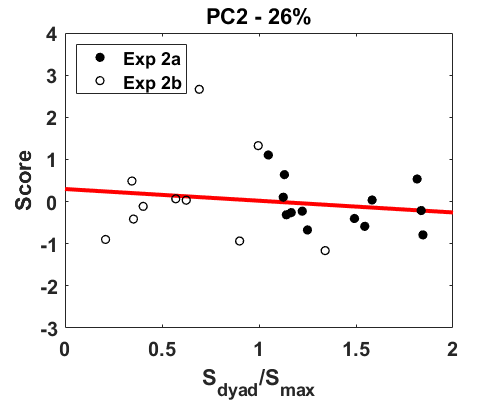* |

*Figure S.3.1*. fPCA of capacity coefficient function. (a) A scree plot showing the amount of variance accounted before varimax rotation for by each eigenfunction, ordered from highest to lowest. (b) Mean capacity function and the principal component function (left panel) and the contrast function (right panel). (c) Plot of the time-based measure (factor score of the first component) against accuracy-based measure (Sdyad/Smax). (d) Plot of the time-based measure (factor score of the second component) against accuracy-based measure (Sdyad/Smax).

| *(a)* 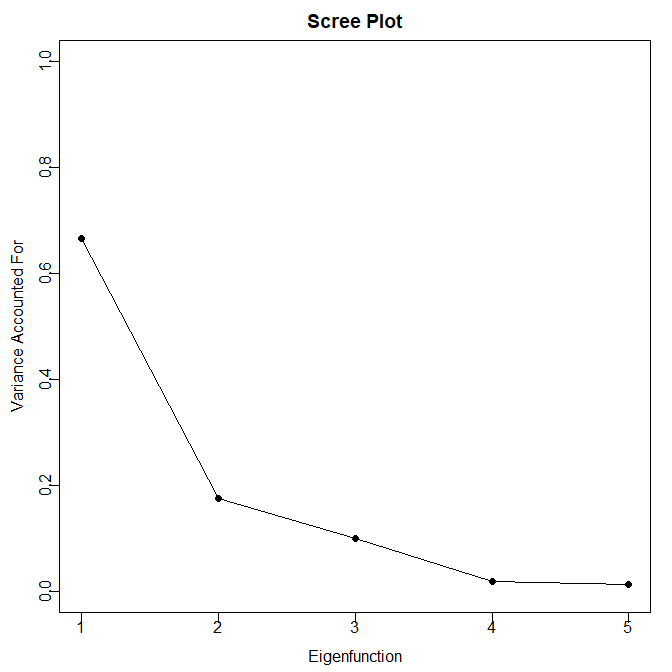 |
| --- |
| *(b)* 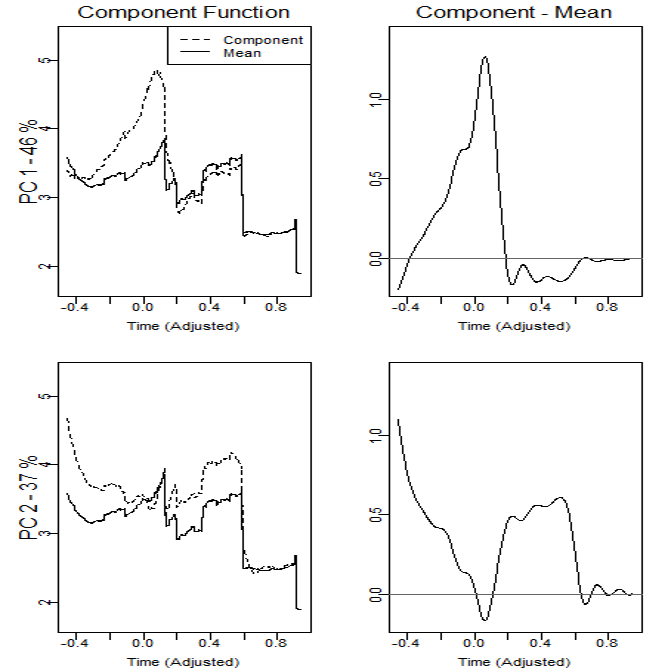 |
| *(c)*  *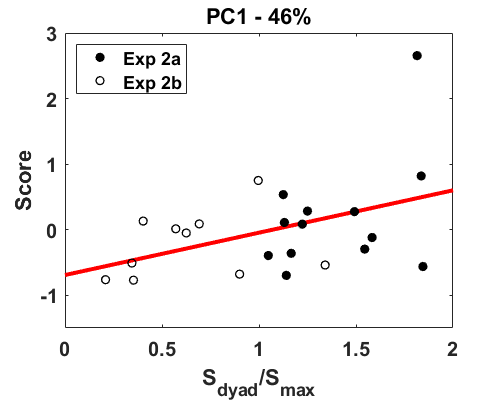* |
| *(d)*  *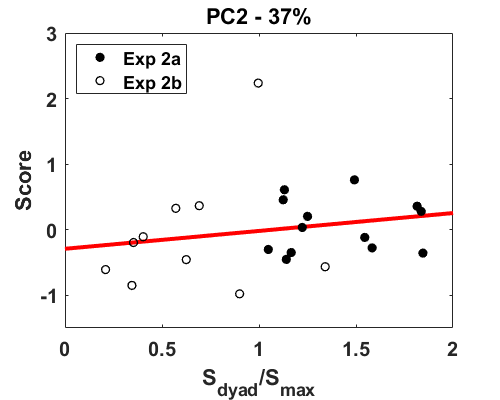* |

*Figure S.3.2*. fPCA of assessment function for correct and fast responses. (a) A scree plot showing the amount of variance before varimax rotation accounted for by each eigenfunction, ordered from highest to lowest. (b) Mean assessment function for correct and fast responses and the principal component function (left panel) and the contrast function (right panel). (c) Plot of the time-based measure (factor score of the first component) against accuracy-based measure (Sdyad/Smax). (d) Plot of the time-based measure (factor score of the second component) against accuracy-based measure (Sdyad/Smax).

| *(a)* 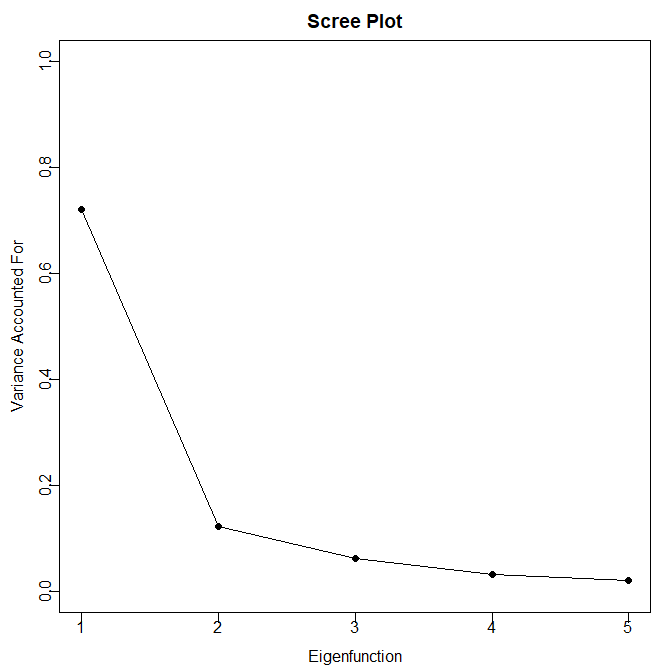 |
| --- |
| *(b)* 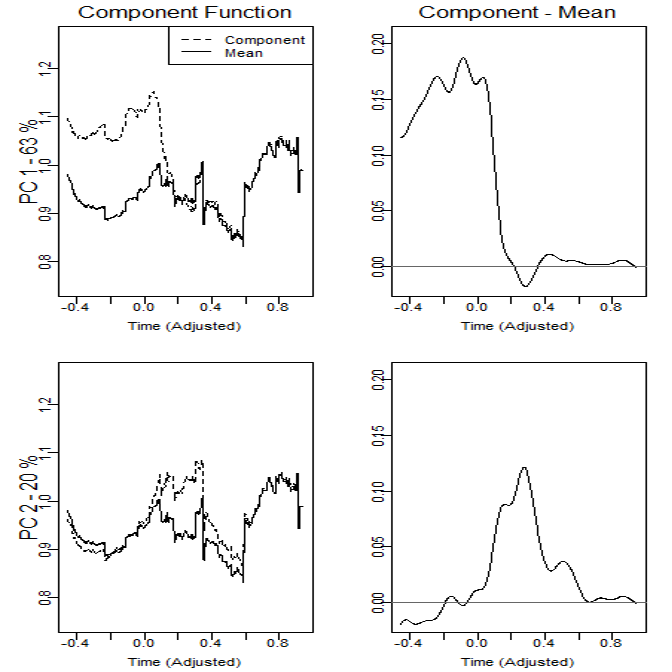 |
| *(c)*  *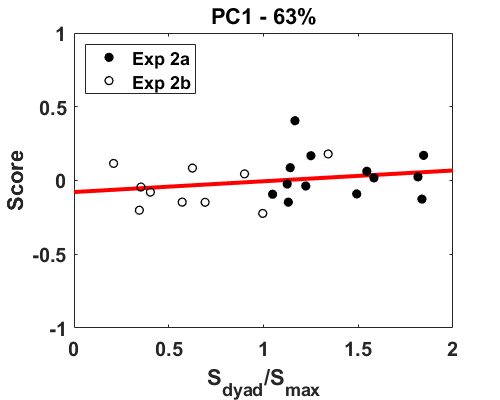* |
| *(d)*  *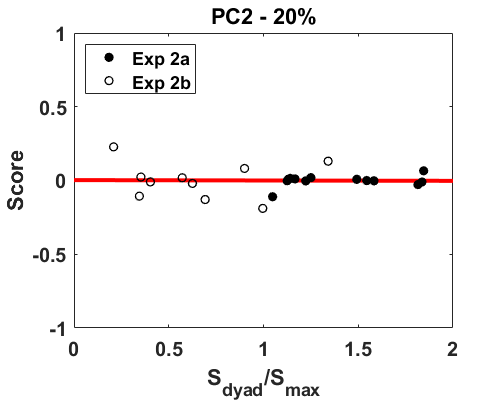* |

*Figure S.2.3*. fPCA of assessment function for correct and slow responses. (a) A scree plot showing the amount of variance before varimax rotation accounted for by each eigenfunction, ordered from highest to lowest. (b) Mean assessment function for correct and slow responses and the principal component function (left panel) and the contrast function (right panel). (c) Plot of the time-based measure (factor score of the first component) against accuracy-based measure (Sdyad/Smax). (d) Plot of the time-based measure (factor score of the second component) against accuracy-based measure (Sdyad/Smax).

| *(a)* 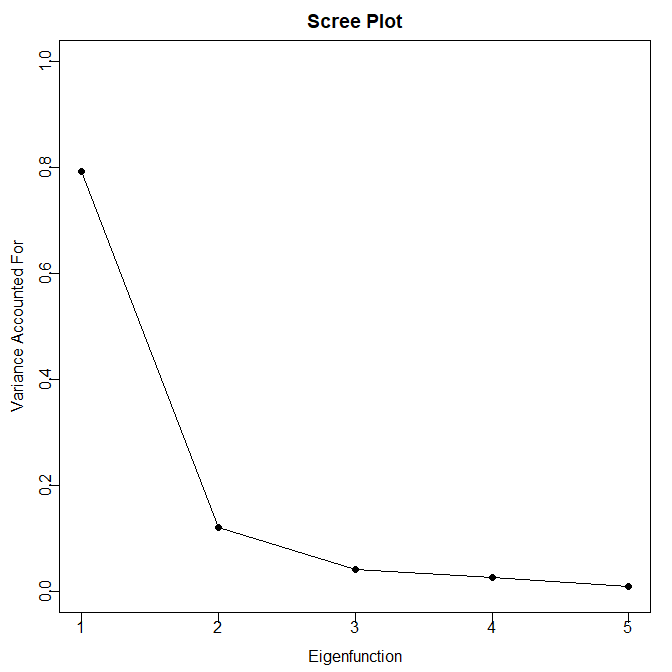 |
| --- |
| *(b)* 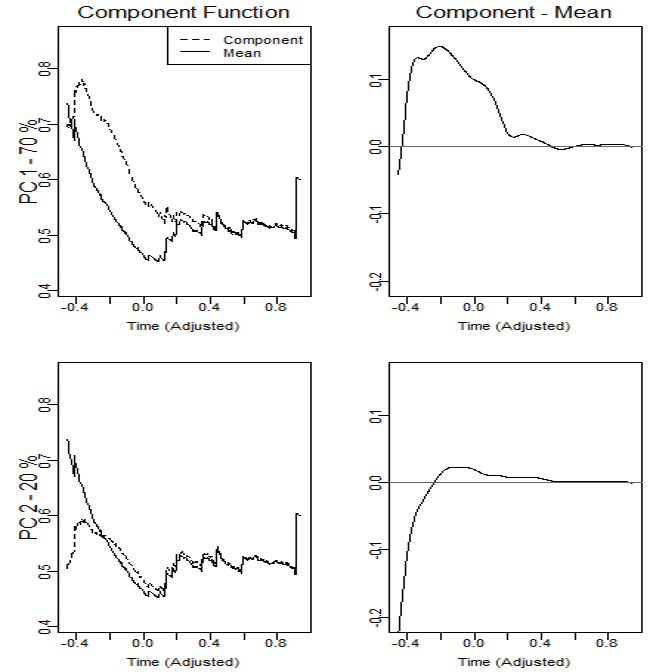 |
| *(c)*  *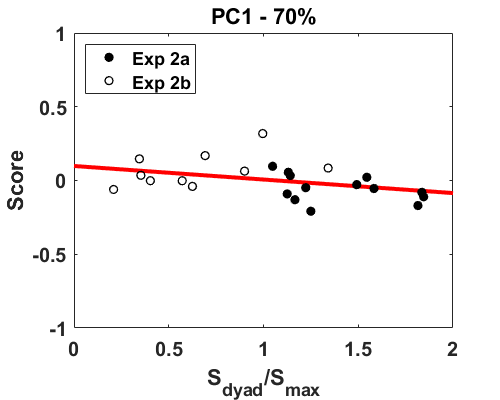* |
| *(d)*  *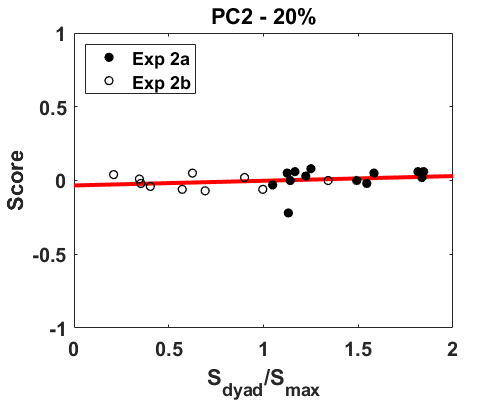* |

*Figure S.3.4*. fPCA of assessment function for incorrect and fast responses. (a) A scree plot showing the amount of variance before varimax rotation accounted for by each eigenfunction, ordered from highest to lowest. (b) Mean assessment function for incorrect and fast responses and the principal component function (left panel) and the contrast function (right panel). (c) Plot of the time-based measure (factor score of the first component) against accuracy-based measure (Sdyad/Smax). (d) Plot of the time-based measure (factor score of the second component) against accuracy-based measure (Sdyad/Smax).

| *(a)* 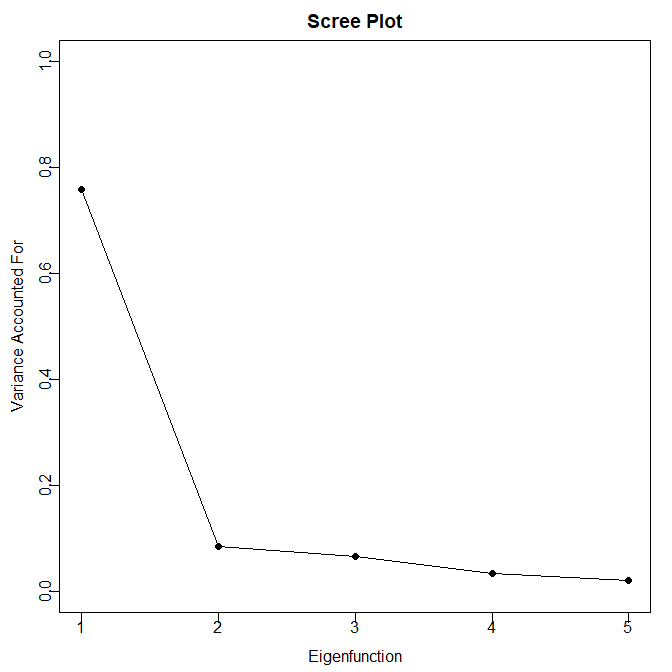 |
| --- |
| *(b)* 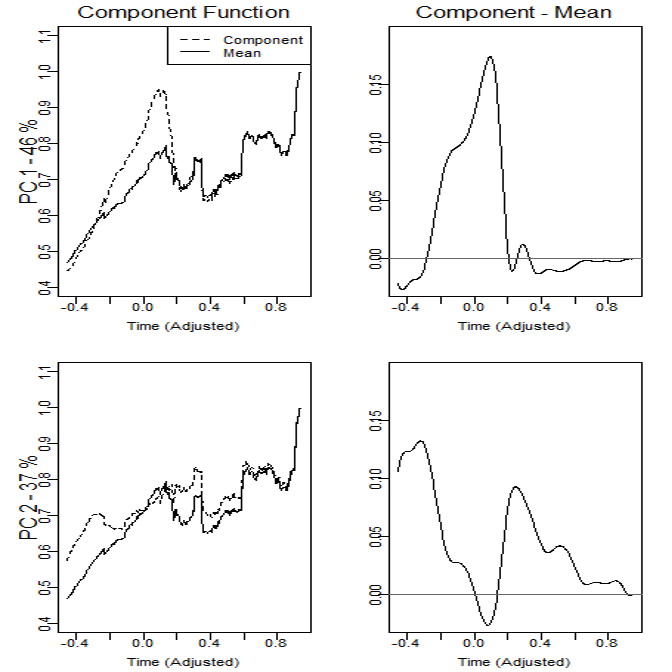 |
| *(c)*  *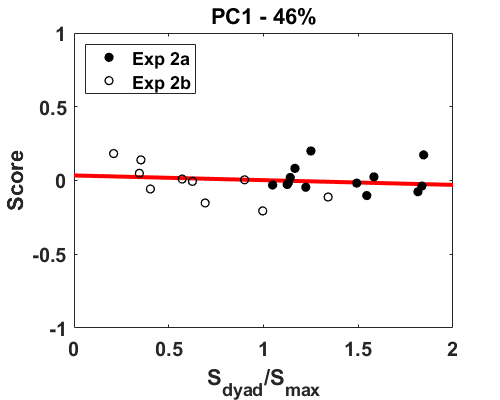* |
| *(d)*  *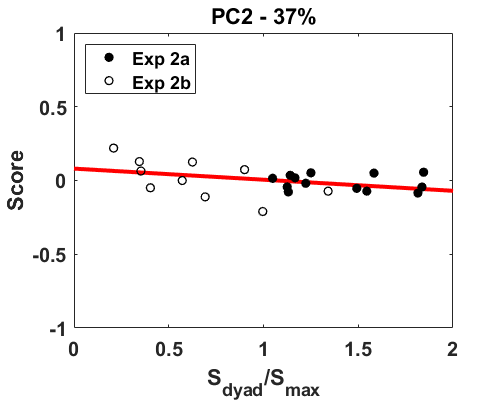* |

*Figure S.3.5*. fPCA of assessment function for incorrect and slow responses. (a) A scree plot showing the amount of variance before varimax rotation accounted for by each eigenfunction, ordered from highest to lowest. (b) Mean assessment function for incorrect and slow responses and the principal component function (left panel) and the contrast function (right panel). (c) Plot of the time-based measure (factor score of the first component) against accuracy-based measure (Sdyad/Smax). (d) Plot of the time-based measure (factor score of the second component) against accuracy-based measure (Sdyad/Smax).
